# Generation of Fermat’s spiral patterns by solutal Marangoni-driven coiling in an aqueous two-phase system

**DOI:** 10.1038/s41467-022-34368-5

**Published:** 2022-11-23

**Authors:** Yang Xiao, Neil M. Ribe, Yage Zhang, Yi Pan, Yang Cao, Ho Cheung Shum

**Affiliations:** 1 Department of Mechanical Engineering, The University of Hong Kong, Pokfulam Road, Hong Kong, China; 2 Lab FAST, University Paris-Saclay, CNRS, Bât. 530, Campus Univ, 91400 Orsay, France; 3 Advanced Biomedical Instrumentation Centre, Hong Kong Science Park, Shatin, New Territories Hong Kong, China

**Keywords:** Fluid dynamics, Physical chemistry

## Abstract

The solutal Marangoni effect is attracting increasing interest because of its fundamental role in many isothermal directional transport processes in fluids, including the Marangoni-driven spreading on liquid surfaces or Marangoni convection within a liquid. Here we report a type of continuous Marangoni transport process resulting from Marangoni-driven spreading and Marangoni convection in an aqueous two-phase system. The interaction between a salt (CaCl_2_) and an anionic surfactant (sodium dodecylbenzenesulfonate) generates surface tension gradients, which drive the transport process. This Marangoni transport consists of the upward transfer of a filament from a droplet located at the bottom of a bulk solution, coiling of the filament near the surface, and formation of Fermat’s spiral patterns on the surface. The bottom-up coiling of the filament, driven by Marangoni convection, may inspire automatic fiber fabrication.

## Abstract (web summary)

In this work, the authors describe a three-dimensional Marangoni transport process in an aqueous two-phase system. Marangoni-driven spreading initiated with salt leads to the formation of Fermat’s spiral patterns, that are of relevance for materials fabrication and microfluidics.

## Introduction

Directional transport processes in fluids are crucial to life in Nature;^1,2^ examples include the transport of ions like Ca^2+^ across cell membranes^1^, and the tactic behaviors of bacteria and motile cells^2^. In particular, Nature exploits surface tension (SFT) gradients to generate directional transport along fluid surfaces and interfaces^1,2^. This is known as the Marangoni effect, which was first identified by James Thomson in 1855 and then studied by Carlo Marangoni^3^. SFT gradients can be generated either by temperature gradients (thermal Marangoni effect) or concentration gradients (solutal Marangoni effect)^4^. The latter effect can be produced by the addition of a surfactant or other solute that affects the SFT. This is a spontaneous isothermal process^5^. The solutal Marangoni effect has been widely used for mass transfer on liquid surfaces, to form different kinds of patterns, such as fingering^6,7^, schlieren patterns^8^, and flower-like patterns^9^. Those patterns are usually liquid films generated by the Marangoni-driven spreading of a droplet on an aqueous substrate^6–10^, which has been applied in the fabrication of ultra-thin hydrogel films^11^, organic thin film transistors^12^, and organic solar cells^13^.

In addition to spreading on an air/liquid interface, Marangoni forces can also generate convective flows in the bulk liquid^14^. This Marangoni convection results in mass transfer from within the bulk solution to the surface. The combination of Marangoni-driven spreading and Marangoni convection represents a type of continuous transport process that has scarcely been reported in the literature. We shall call this process “Marangoni transport”.

**Fig. 1:**
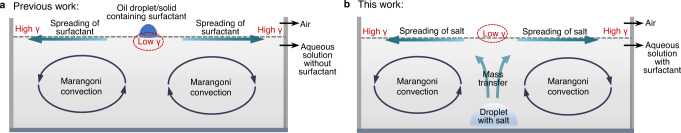
Mass transfer based on solutal Marangoni effect. **a** Generation of SFT (γ) gradients by the addition of an oil droplet or a solid containing surfactant to an surface of the aqueous solution, and the resulting Marangoni-driven spreading and Marangoni convection. **b** Marangoni transport from a droplet to the bulk solution surface (by Marangoni convection), and then at the air/water interface (by Marangoni effect).

Marangoni transport requires the generation and maintenance of SFT gradients. One traditional way to produce such gradients is to deposit a surfactant like alcohol or camphor on the surface of an aqueous solution (Fig. 1a)^5,8^. While the release of surfactant from the deposited droplet or solid particle reduces the SFT efficiently, the release tends to be asymmetric, generating self-motion of the droplet/particle that disturbs the transport process^15^. Moreover, the surfactant tends to remain at the surface and to prevent further decrease of the SFT, making the transport process difficult to control^6–9^.

In this paper, we explore another way to generate SFT gradients, namely by adding salt to a solution containing surfactant. The basis of this procedure is the fact that the SFT of an ionic surfactant solution can be strongly affected by the surfactant-counterion interaction^16,17^. The added salt binds to the existing surfactant, further decreasing the SFT^16,17^. On this basis, we propose a method to produce SFT gradients and to generate continuous transport from a bulk solution to the surface and then along that surface (Fig. 1b). To achieve this, we use an aqueous two-phase system (ATPS) based on polyethylene glycol (PEG) and dextran (DEX), which can maintain a continuous transfer of salt between the two phases^18–20^. The salt source is a DEX-rich droplet at the bottom of a PEG-rich solution with an anionic surfactant. The salt can electrostatically interact with the anionic surfactant on the bulk solution surface to generate SFT gradients and Marangoni convection (Fig. 1b). Consequently, a colloidal filament forms at the top of the droplet, moves upward, coils near the bulk solution surface, and spreads along the surface to form Fermat’s spiral patterns. These patterns can be continuously generated and maintained by the Marangoni transport system. Our system has potential applications in areas such as microfluidics, materials fabrication, cargo transport, and heavy metal collection.

## Results and discussion

### Observation of Marangoni convection

**Fig. 2:**
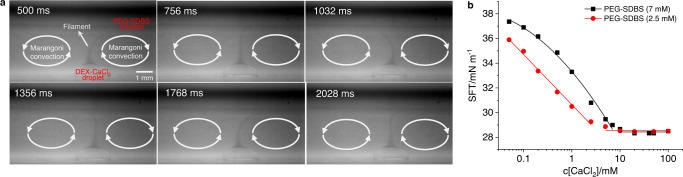
Generation of Marangoni convection by CaCl_2_ induced SFT decrease of SDBS solution. **a** Generation of Marangoni convection (indicated by white ovals) by the addition of a droplet of DEX-CaCl_2_ (100 mM) to a solution of PEG-SDBS (7 mM) in a glass cuvette (24 × 24 × 24 mm). Hollow glass beads (diameter 8–12 μm, density 1.05–1.15 g/cm^3^) were added to the PEG-SDBS solution to visualize the flow. Images were taken from Supplementary Movie 1. **b** Black line: SFT vs. c[CaCl_2_] for PEG-SDBS (7 mM). SFT with no CaCl_2_ is 37.9 mN/m. Red line: same, but for PEG-SDBS (2.5 mM). SFT with no CaCl_2_ is 37.5 mN/m.

The PEG and DEX-based ATPS was prepared according to the procedure described in the Methods section (Supplementary Fig. 1 and Supplementary Table 1)^21^. The upper PEG-rich (~ 8% w/w) layer was used as the bulk solution after the addition of a surfactant, sodium dodecylbenzene sulfonate (SDBS). The concentration of SDBS was initially set as 7 mM, since the surfactant solution was relatively stable at this concentration^22^. The DEX-rich (~16% w/w) solution with CaCl_2_ (100 mM) served as the source of droplets, which were deposited by autopipette at the bottom of a container filled with PEG-SDBS solution. After the pipette was withdrawn, Marangoni convection in the bulk solution was observed (Supplementary Movie 1). As the movie shows, the flow forms a closed loop from the DEX-CaCl_2_ droplet to the air-water interface of the PEG-SDBS solution, then away from the droplet near the surface, and finally back towards the droplet along the bottom. The resulting Marangoni convection exhibited periodic oscillation, which was probably due to the asymmetric distribution of CaCl_2_ on the surface. A filament of fluid from the droplet rose continuously to the surface, and oscillated with a frequency of around 1 Hz (Fig. 2a). Decreasing the concentration of CaCl_2_ from 100 mM to 50 mM had almost no effect on the Marangoni convection, so 50 mM of CaCl_2_ was used in the DEX-rich droplet in the following experiments unless specified.

Eliminating either CaCl_2_ or SDBS from the system suppressed convection entirely, indicating that the decrease of SFT by the interaction between CaCl_2_ and SDBS played a crucial role in generating Marangoni convection. This was further confirmed by the SFT measurement. Addition of CaCl_2_ (100 mM) to a PEG-SDBS (c[SDBS] = 7 mM) solution decreases the SFT from 37.9 to 28.5 mN/m (Supplementary Table 2B), consistent with the reported trend^16,17^. The SFT vs. c[CaCl_2_] (logarithmic scale) curve decreases first (c[CaCl_2_] ≤ 7 mM), and then almost stays constant (c[CaCl_2_] ≥ 10 mM) (black line in Fig. 2b). The maximum SFT difference (ΔSFT) that we could achieve is, therefore, 9.5 mN/m by adding a DEX-CaCl_2_ (c[CaCl_2_] = 50 or 100 mM) droplet to PEG-SDBS (7 mM) solution. A similar trend is observed for a PEG-SDBS solution containing 2.5 mM of SDBS (red line in Fig. 2b), and the maximum ΔSFT is 8.9 mN/m. Yet although the decrease of SFT by the interaction of CaCl_2_ and SDBS has been investigated, the generation of Marangoni convection by this interaction has not yet been reported to our knowledge.

### Coiling-induced spiral patterns

**Fig. 3:**
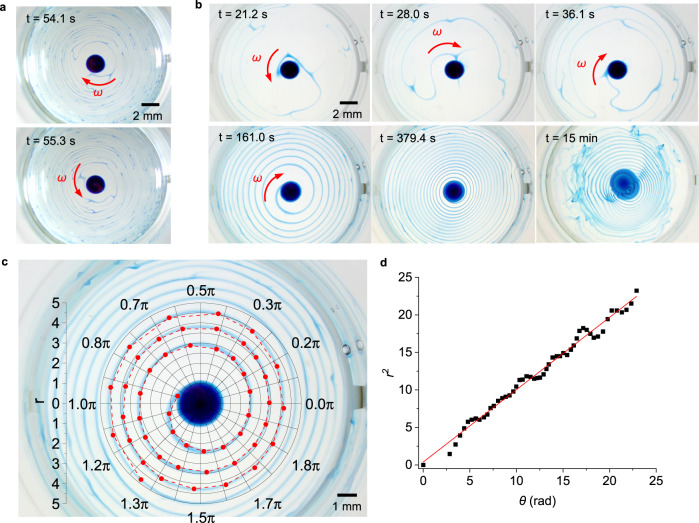
Formation and calculation of Fermat’s spiral patterns. **a** The formation of maze-like patterns by random rotation switching between clockwise and counterclockwise after the addition of a DEX-MB-CaCl_2_ (50 mM) droplet to a round cell (diameter 15.8 mm) filled with PEG-SDBS (7 mM) solution. The red arrows indicate the rotation direction of the filament. Images were obtained from Supplementary Movie 2. **b** Surface patterns as a function of time after the addition of a DEX-MB-CaCl_2_ (50 mM) droplet to a cell filled with PEG-SDBS solution containing 2.5 mM of SDBS: random rotation (*t* = 21.2 and 28.0 s), directional rotation and spiral formation (*t* = 36.1 (start), 161.0 and 379.4 s (termination)), and deformation of the spiral patterns (*t* = 15 min). Images were obtained from Supplementary Movie 3. **c** Polar coordinates (*r*, *θ*) of selected points on the spiral at *t* = 161.0 s. **d** Plot of *r*^2^ vs. *θ* for the same spiral.

To investigate the mass transfer process further, we added methylene blue dye (MB; 1% w/w) to the DEX-rich solution with 50 mM CaCl_2_. A small droplet (1 μL) of this solution was then deposited on the bottom of a round cell (diameter 15.8 mm) filled with the PEG-SDBS (7 mM) solution (depth 2.5 mm, volume 0.5 mL) described previously. The rising filament started to rotate near the surface of the bulk solution. The rotation switched randomly between clockwise and counterclockwise. As a result, maze-like patterns formed on the surface (Fig. 3a, Supplementary Movie 2). The filament appeared red under white light, probably due to light scattering (Supplementary Movie 2). The rotation frequency of the filament was around 1 Hz. Because the rotation was fast and switched randomly between clockwise and counterclockwise, the pattern on the surface was complex and unpredictable.

When the concentration of SDBS in the PEG-SDBS solution was reduced to 2.5 mM, the rotation frequency diminished to less than 0.2 Hz. The filament first rotated randomly between clockwise and counterclockwise for a few seconds (*t* = 0 – 36.1 s in Supplementary Movie 3) and then only clockwise (*t* = 36.1–379.4 s, Fig. 3b and Supplementary Movie 3). As a result, spiral patterns formed on the surface once the filament reached the surface (Fig. 3b, Supplementary Movie 3). The direction of rotation of the spiral has almost an equal probability to be clockwise or anticlockwise (Supplementary Fig. 2), indicating that the selection of the spirality likely depends on the initial random rotation^23^. After the rotation ended at *t* = 379.4 s, the patterns slowly deformed over about 10 min due to the diffusion of the materials in the blue spiral stripe at the surface (*t* = 15 min, Fig. 3b). To quantify the spiral patterns, we determined the relation between the radius (*r*) and azimuth (*θ*) for the pattern at *t* = 161.0 s (Fig. 3c). The resulting equation was *r*^2^ = 0.38 + 0.96*θ*, with an Adjusted R-squared of 0.986, indicating an excellent fit (Fig. 3d). When *θ* > π, the constant 0.38 becomes negligible and the equation simplifies to *r*^2^ = 0.96*θ*. This is just the equation of Fermat’s spiral, *r*^2^ = a^2^*θ* (a^2^ = 0.96)^24^. Similarly, we obtained a series of a^2^ by analyzing the spiral patterns at different times (supplementary Fig. 3a, b). We found that the value of a^2^ decreases with time (Supplementary Fig. 3c) because of the constant formation of stripes on the limited surface. To the best of our knowledge, similar case, in which Fermat’s spiral is generated automatically by Marangoni convection in a non-equilibrium system, has not been reported in the literature.

### Composition of the filament and spiral patterns

**Fig. 4:**
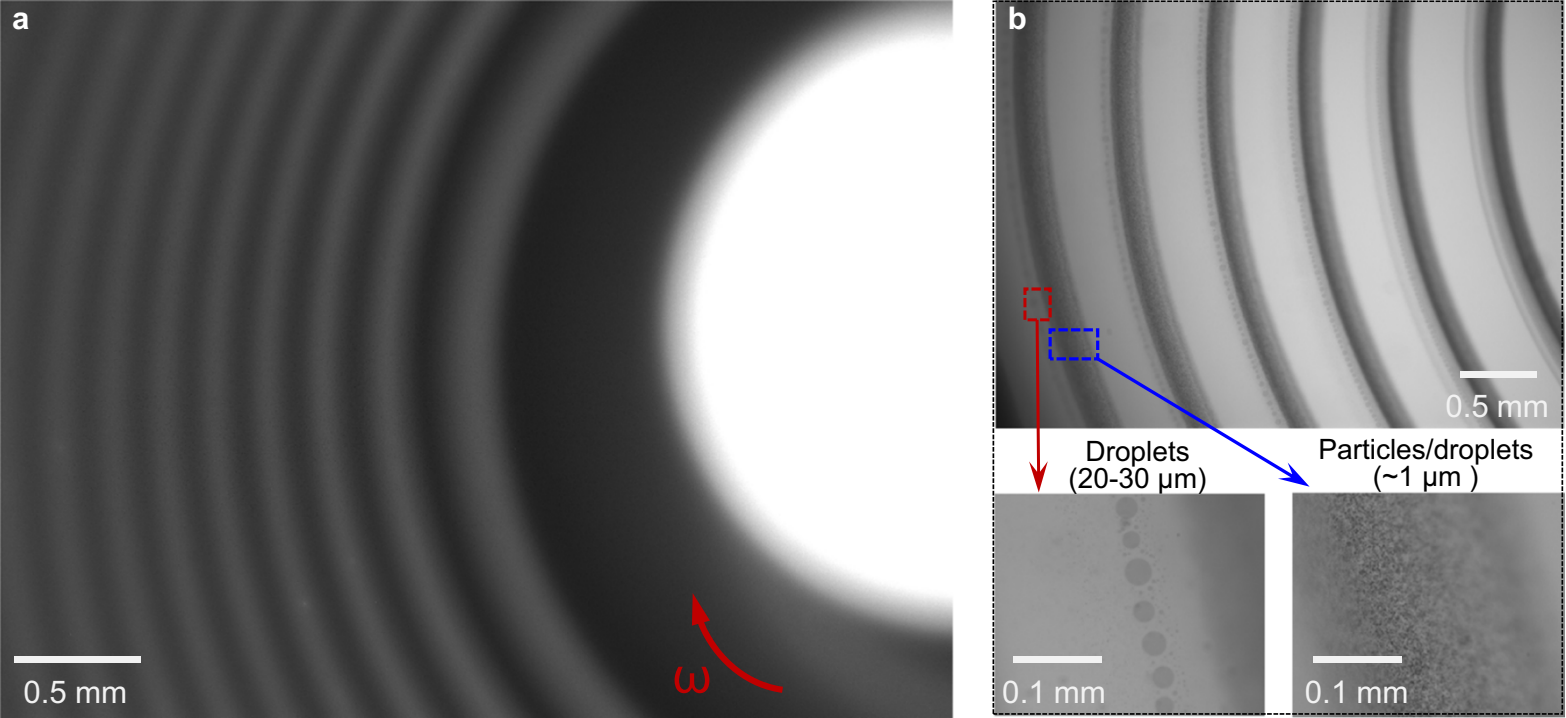
Composition of the filament and spiral patterns. **a** Formation of spiral patterns by the addition of a 1 μL DEX-CaCl_2_ (50 mM) droplet with Rhodamine 6G (0.05%) to a round cell (diameter 15.8 mm) filled with PEG-SDBS (2.5 mM) solution (depth 2.5 mm). The photo was taken under a fluorescent microscope. **b** Composition of the filament/stripe: Ca(DBS)_2_-PEG-MB particles (diameter ~1 μm) and DEX-MB-CaCl_2_ droplets (diameter ~1 μm). Photos were taken under a microscope.

Since PEG and DEX were used to construct the ATPS, two immiscible phases of the droplet and the bulk solution cannot be formed without PEG or DEX in our system, which could affect the Marangoni transport process. For the system without DEX, the MB-CaCl_2_ (50 mM) drop floated on PEG-SDBS solution and spread rapidly on the surface. While for the system without PEG, mass transfer from a DEX-MB-CaCl_2_ (50 mM) drop to the surface of SDBS (2.5 mM) solution could still be seen, but no spiral patterns formed on the surface. MB was used to visualize the spiral patterns and the patterns could not be seen if its concentration was too low (<0.1% w/w). We also used another cationic dye Rhodamine 6G (0.05% w/w) instead of MB. Spiral patterns were then observed under a fluorescence microscope (Fig. 4a), indicating that the dye tends to stay within the filament/stripe, and that the concentration of the dye can be relatively low. Further experiments show that the spiral patterns include small particles and droplets. In our system, negatively charged micelles are formed in the PEG-SDBS solution^25,26^, which could capture Ca^2+^ (>5 mM) to form colloids or precipitates through electrostatic interaction^16,17^. We found that the precipitates are calcium dodecylbenzene sulfonate (Ca(DBS)_2_) with a small amount of PEG (~ 7% w/w, or ~ 0.1% mole/mole) by proton nuclear magnetic resonance (^1^H NMR) spectroscopy, Fourier-transform infrared (FT-IR) spectroscopy and calcium colorimetric assay (Methods section). The blue color of the filament indicates that MB is also absorbed by the micelles^27^. So the particles in the filament consist of Ca(DBS)_2_, PEG and MB. While the filament spread on the surface, tiny droplets (diameter 20-30 μm) were seen in front of the spiral stripe (Fig. 4b), which were probably generated by the coalescence of the microdroplets (diameter ~ 1 μm) in the filament. We, therefore, conclude that the spiral patterns consist of Ca(DBS)_2_-PEG-MB particles and DEX-MB-CaCl_2_ droplets. White light directed onto the filament from top left/right is scattered by the particles and droplets so that the filament appears red (Supplementary Movie 4).

### Characterization of spiral patterns

**Fig. 5:**
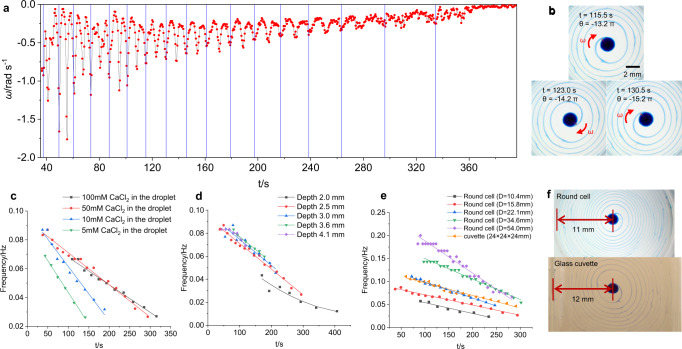
Effect of CaCl_2_ concentration in the droplet, solution depth, and container size on the frequency of spiral patterns. **a** Angular velocity curve *ω*(*t*) obtained by tracking the rotation of the filament in a round cell (diameter 15.8 mm) filled with PEG-SDBS (2.5 mM) solution (depth 2.5 mm) (Supplementary Movie 3). The *ω* data were recorded every 0.5 s. *ω* < 0 because the rotation was clockwise. **b** Corresponding images for the three peaks in the *ω*(*t*) curve at 115.5 (*θ* = −13.2 π*)*, 123.0 (*θ* = −14.2 π*)*, and 130.5 s (*θ* = −15.2 π*)*, respectively. **c** *f*_*r*_(*t*) curve measured for the addition of a 1 μL droplet of DEX-MB-CaCl_2_ containing 100 (black line), 50 (red line), 10 (blue line), and 5 (green line) mM of CaCl_2_ to a round cell (diameter 15.8 mm) filled with PEG-SDBS (2.5 mM) solution (depth 2.5 mm), respectively. **d** *f*_*r*_(*t*) curves obtained for spiral patterns in a round cell (diameter 15.8 mm) with a solution depth of 2.0 (black line), 2.5 (red line), 3.0 (blue line), 3.6 (green line), and 4.1 mm (purple line), respectively. The curves for 2.0 and 4.1 mm were fitted by second-order polynomials and the rest by straight lines. **e** Average frequency *f*_*r*_(*t*) as a function of time for spiral patterns in round cells (solution depth 2.5 mm) with a diameter (D) of 10.4 (black line), 15.8 (red line), 22.1 (blue line), 34.6 (green line), and 54.0 mm (purple line), and a glass cuvette (24 × 24 × 24 mm) (orange line), respectively. Straight-line fits to the curves are shown. **f** The distance from the droplet to the wall in the glass cuvette and the 22 mm diameter round cell.

The time-varying angular speed *ω*(*t*) of rotation of the spiral patterns was obtained by analyzing Supplementary Movie 3 using the software Tracker. Figure 5a shows that peak values of *ω* occur periodically. The distance between two peaks is the time required for the filament to make a half turn (π) (Fig. 5b). The average frequency *f*_*r*_ is the reciprocal of the time period of three peaks. The *f*_*r*_(*t*) curve shows a linear decrease with time (red line, Fig. 5c). We also notice that a phase shift of 0.3 π exists between the angular speed vs. angle curve (*ω*(*θ*)) and the filament size near the spiral core (on the surface above the droplet) vs. angle curve (*S*(*θ*)) (Supplementary Fig. 4, Supplementary Movie 4), indicating that the oscillation of the angular speed can be attributed to the periodic accumulation of the filament on the surface and the subsequent spreading.

Since CaCl_2_ is crucial for decreasing the SFT of the PEG-SDBS solution, we firstly investigated the effect of CaCl_2_ concentration in the DEX-MB-CaCl_2_ droplet on the spiral patterns. When the concentration of CaCl_2_ in the droplet was 50 or 100 mM, the corresponding *f*_*r*_(*t*) curves nearly coincided (Fig. 5c), indicating that 50 mM of CaCl_2_ in the droplet was able to provide sufficient CaCl_2_ to decrease SFT. However, for droplets with 5 and 10 mM of CaCl_2_, the average frequency *f*_*r*_ was much lower and decreased faster with time than that with 50 mM of CaCl_2_. When the concentration of CaCl_2_ in the droplet was 1 mM, the spiral just rotated a half turn. If the concentration of CaCl_2_ was less than 1 mM, no spiral pattern formed. The results show that the system is fed by the DEX-CaCl_2_ droplet and can be directly controlled by the concentration of CaCl_2_ in the droplet.

We also investigated the influence of depth on our spiral patterns^15^. To generate such patterns in a cell 15.8 mm in diameter, the depth of the bulk solution had to be between 1.5 and 4.8 mm. For depths >4.8 mm, the initial Marangoni convection was not strong enough to maintain mass transfer between the droplet and the surface. For depths between 2.5 and 4.1 mm, the average frequency did not change much with depth, indicating that depth in this range did not affect the surface distribution of SFT gradients. However, the persistence time decreased with increasing depth in this range (Fig. 5d), probably because the depth affected the supply of CaCl_2_ from the droplet to the surface. However, at shallower depths (e.g., 2.0 mm), the surface SFT gradients were affected, leading to a decrease in average frequency relative to the values obtained at greater depths (Fig. 5d). When the depth was below 1.5 mm, the droplet touched the surface of the bulk solution. In this case, fluid was released randomly from the droplet, and no spiral patterns were formed.

Similar spiral patterns and linear *f*_*r*_(*t*) curves were obtained for cells with the same depth (2.5 mm) but different diameters (10.4, 15.8, 22.1, 34.6, and 54.0 mm) (Fig. 5e). The average frequency is higher for larger cells, as shown in Fig. 5e, which facilitates the spread of CaCl_2_ on the surface, slowing down the accumulation of CaCl_2_ and maintaining a higher SFT gradient. Consequently, spiral patterns could be generated with a higher average frequency. We also performed experiments in a square glass cuvette (24 × 24 × 24 mm). After the addition of a DEX-MB-CaCl_2_ droplet (c[CaCl_2_] =  50 mM, 1 μL) to the 2.5 mm depth of PEG-SDBS (2.5 mM) solution, a *ω*(*t*) curve was obtained. This curve shows that peaks appeared approximately every half revolution, similarly to what we saw for a 15.8 mm diameter round cell. However, the maximum value of *ω* was much higher and decreased only slightly with time (Supplementary Fig. 5). The average frequency *f*_*r*_(*t*) also decreased linearly with time (orange line, Fig. 5e). The *f*_*r*_(*t*) curve obtained using this glass cuvette was similar to that obtained using a 22.1 mm round cell (Fig. 5f), probably because the distance between the filament and the wall (12 mm for the cuvette and 11 mm for the round cell) was almost the same in both cases (Fig. 5f). As a result, the SFT gradients, which provided the driving force for the spreading of spiral patterns on the surface, were also similar in both cases. We also found that the spiral patterns can still be generated in containers with other shapes, for example, a narrow rectangular cuvette (5 × 100 × 10 mm), but the patterns might be deformed accordingly (Supplementary Fig. 6).

**Fig. 6:**
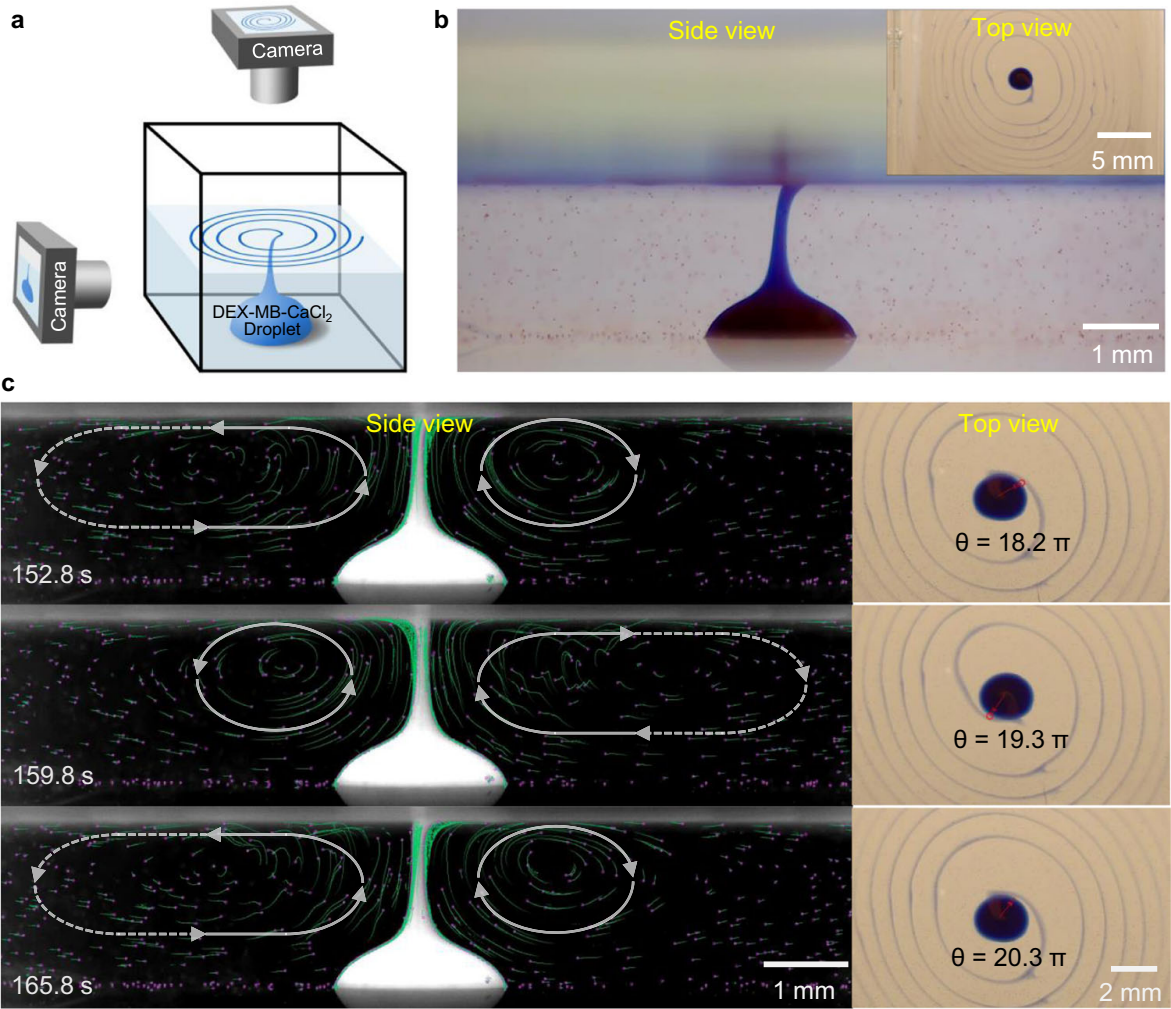
Side view of the Marangoni transport process. **a** Schematic of apparatus used to capture the Marangoni convection and the spiral patterns formed by the deposition of a DEX-MB-CaCl_2_ (50 mM) droplet in a glass cuvette (24 × 24 × 24 mm) filled with PEG-SDBS (2.5 mM) solution (depth 2.5 mm). Cameras were fixed in the side and top directions to take movies beginning when the droplet was added to the bulk solution. **b** Coiling (side view) and spiral pattern formation (top view) subsequent to the deposition of the DEX-MB-CaCl_2_ droplet. PS particles (diameter 22 μm; red dots) were added to the solution to visualize the convection. **c** Asymmetric Marangoni convection around the filament (left) and the corresponding spiral patterns (right) at 152.8, 159.8, and 165.8 s. Peaks of the *ω*(*t*) curve occurred at those three times. The green lines are trajectories of the PS particles (diameter 22 μm; pink dots) over 5 s. White ovals indicate the direction of the Marangoni circulation.

The use of a glass cuvette made it possible to obtain side views of the mass transfer process (Fig. 6a). A blue filament stretching from the droplet to the surface of the bulk solution was formed, and coiled once it came close to the surface (Fig. 6b). This process was superficially similar to liquid rope coiling driven by gravity, except that here the filament moves upward against gravity^28^. The filament rotated randomly between clockwise and counterclockwise at the beginning. After around 44 s, the filament rotated counterclockwise, and spiral patterns started to form on the surface (Fig. 6b, Supplementary Movie 5). To visualize the flow, we tracked the trajectories of red polystyrene (PS) particles (diameter 22 μm, density 1.05 g/cm^3^) added to the PEG-SDBS solution (Fig. 6c, Supplementary Movie 6). An asymmetric flow around the filament was observed (Fig. 6c). This is probably because that CaCl_2_ in the filament could diffuse to the surrounding surface to further decrease SFT and increase SFT gradients near the filament, leading to stronger Marangoni convection underneath the stripe. The asymmetric flow may in turn contribute to the filament’s oscillation^29^. The visualization also showed that the filament’s velocity was similar to that of the outer fluid, further indicating that the filament was advected upward by the Marangoni convection.

### Mechanism

#### Interactions between the surfactant and the solutes

Although both surfactant-polymer interaction and surfactant-counterion interaction are involved in our system, the SFT decrease mainly depends on the latter. In the PEG-SDBS bulk solution, the nonionic polymer PEG could influence the properties of the anionic surfactant SDBS through the interactions between the polymer chain and the dodecylbenzene sulfonate (DBS^-^) headgroup^25,26^. This kind of polymer-surfactant interaction is relatively weak^25,26^. An SFT difference of around 2.3 mN/m was observed for the SDBS solution (2.5 mM) with or without PEG (~8%), and 4.4 mN/m for the SDBS solution (7 mM) with or without PEG (~8%) (Supplementary Table 2, Supplementary Fig. 7). CaCl_2_ could affect SFT of the PEG-SDBS solution through the electrostatic interaction between the divalent cation Ca^2+^ and DBS^-^^16,17^. Ca^2+^ could bind strongly to the benzenesulfonate headgroup to screen the electrostatic repulsion between the surfactant headgroups on the surface, leading to a significant increase in surfactant adsorption^16^. As a result, even a relatively low concentration of Ca^2+^ could cause a dramatic reduction in SFT of the PEG-SDBS solution (Fig. 2b). This binding has a stronger effect on the SFT of the PEG-SDBS solution with a lower concentration of SDBS (Fig. 2b), because less Ca^2+^ is bound by SDBS in the solution, and more Ca^2+^ is available to interact with the surfactant at the surface adsorption layer^16,17^. However, the binding between Ca^2+^ and SDBS is so strong that a further increase in the concentration of Ca^2+^ (>5 mM) will result in the precipitation of Ca(DBS)_2_ (Supplementary Fig. 8), thus preventing a further decrease in SFT (Fig. 2b). The surfactant-counterion interaction is much stronger than the surfactant-polymer interaction, and therefore dominates the SFT decrease. This is further demonstrated by the plot of SFT against c[CaCl_2_] for SDBS solution without PEG, which shows a similar trend as that of PEG-SDBS solution (Supplementary Fig. 7).

#### Initiation and maintenance of the Marangoni transport system

**Fig. 7:**
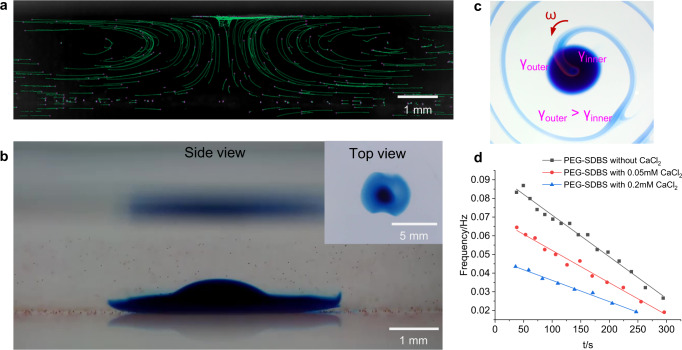
Initiation of the Marangoni transport system and rotation of the filament. **a** Marangoni convection induced by the deposition of a tiny amount of DEX-MB-CaCl_2_ (<0.05 μL) on the surface of a PEG-SDBS solution (2.5 mM) containing tracer particles (diameter 22 μm) in a glass cuvette. The green lines are the trajectories of the PS particles during 5 s. The pink dots indicate the current positions of the PS particles. **b** Diffusion of MB and CaCl_2_ from a DEX-MB-CaCl_2_ droplet (1 μL) that was carefully deposited at the bottom of the PEG-SDBS (2.5 mM) bulk solution. **c** Schematic of the surface tension difference while the spiral pattern is rotating. **d** Time-varying average frequency *f*_*r*_(*t*) determined from the spiral patterns produced by the addition of a droplet of DEX-MB-CaCl_2_ (50 mM) to a cell (diameter 15.8 mm) filled with PEG-SDBS (50 mM) solution containing 0, 0.05, and 0.2 mM of CaCl_2_, respectively.

In our experiments, Marangoni convection is triggered by the residue of the DEX-MB-CaCl_2_ droplet on the pipette tip, which decreases the SFT (Fig. 1b). When we added a tiny amount of DEX-MB-CaCl_2_ (<0.05 μL) to the surface of a PEG-SDBS (2.5 mM) solution, convection underneath the droplet occurred immediately (Fig. 7a, Supplementary Movie 7), indicating that a tiny amount of CaCl_2_ on the air/water interface is enough to generate Marangoni convection. If we deposited a DEX-MB-CaCl_2_ droplet (1 μL) beneath the PEG-SDBS solution carefully, and then took the pipette tip out of the solution slowly, the contact of CaCl_2_ residue on the tip with the bulk solution surface could be avoided. As a result, no convection or blue filament was observed in the bulk solution at first. However, once a pipette tip with a tiny amount of DEX-MB-CaCl_2_, DEX-CaCl_2_, PEG-CaCl_2_, or pure CaCl_2_ solution touched the aqueous surface above the droplet, the convection and blue filament were generated immediately (Supplementary Movie 8). The tip with only DEX, DEX-MB, or PEG solution, cannot trigger the transport process, indicating that they are not able to efficiently decrease the SFT of the solution (Supplementary Table 2A). We also used a tip with a tiny amount of ethanol (SFT = 23.6 mN/m) instead, and the system could be initiated, further confirming that SFT decrease is necessary.

Once the Marangoni convection starts, CaCl_2_ in the DEX-MB-CaCl_2_ droplet on the bottom is transferred to the surface by the flow, thereby maintaining continuous circulation. Since the diffusion rate of CaCl_2_ is extremely slow^30^, if we generated a DEX-MB-CaCl_2_ droplet under the PEG-SDBS solution carefully without inducing Marangoni convection, it took 8 minutes for CaCl_2_ to diffuse from the droplet to the bulk solution surface and initiate the Marangoni transport (Supplementary Movie 9). Before the initiation, the blue materials formed surrounding the droplet and became flat due to gravity (Fig. 7b), indicating that they have a slightly higher density than the bulk solution. The results show that both the initiation and maintenance of the system are due to Marangoni convection.

### Formation of spiral patterns

When the filament reaches the surface of the PEG-SDBS solution by Marangoni convection, it starts to rotate. The surface tension gap between the inner side (near the spiral core, γ_inner_) and the outer side (γ_outer_) of the filament is the origin of the rotation (Fig. 7c). Since the inner side is closer to the spiral core, the concentration of CaCl_2_ is higher than the outer side. As a result, γ_inner_ is lower than γ_outer_, thus pushing the filament around. Moreover, CaCl_2_ in the filament could also diffuse to the surrounding solution to increase the SFT gradient and facilitate the spreading. Consequently, as the filament is rotating on the surface, some CaCl_2_ would remain behind the filament, thus keeping γ_inner_ at a relatively low level and therefore preventing the filament from rotating back. To prove our hypothesis, we decreased γ_outer_ by adding a PEG-SDBS (2.5 mM) drop with CaCl_2_ (0.5 mM) to the outer area while the filament was rotating clockwise. Consequently, the rotation direction reversed to counterclockwise (Supplementary Movie 10). As a control, we added a PEG-SDBS (2.5 mM) drop without CaCl_2_ to the same area, and the rotation was not affected. Similarly, at the beginning when DEX-MB-CaCl_2_ is just added to the PEG-SDBS solution, the filament contains a large amount of CaCl_2_ and CaCl_2_ diffuses from the filament to the bulk solution. Consequently, γ_inner_ is close to γ_outer_ and random oscillation of the spiral patterns on the bulk solution surface can be observed.

Because CaCl_2_ is continually being lost from the droplet and the filament, the amount of CaCl_2_ transferred to the surface is also diminishing. On the other hand, CaCl_2_ accumulates at the surface, which would decrease the SFT. Consequently, the concentration difference between the filament and its surrounding surface keeps decreasing. This is also the reason why the average rotational speed decreases with time. At long times, the concentration difference is not able to create sufficient SFT gradients, leading to the termination of the whole mass transfer process. To investigate further the effect of CaCl_2_ accumulation on SFT gradients, CaCl_2_ (0, 0.05, and 0.2 mM) was added to the PEG-SDBS solution (depth 2.5 mm) in a round cell (diameter 15.8 mm), before the addition of the DEX-MB-CaCl_2_ droplet. The *f*_*r*_(*t*) curve shows that the additional CaCl_2_ decreases the average frequency (Fig. 7d). If the concentration of CaCl_2_ in the bulk solution was higher than 1 mM, no spiral patterns were formed after the addition of the DEX-MB-CaCl_2_ droplet. The SFT of the PEG-SDBS (2.5 mM) solution with 1 mM of CaCl_2_ is 30.5 mN/m (Fig. 2b and Supplementary Table 2D). As we noted above, the minimum SFT of a PEG-SDBS (2.5 mM) solution that could be achieved by the addition of CaCl_2_ (≥ 5 mM) is 28.6 mN/m (Fig. 2b and Supplementary Table 2D). So we estimate the minimum ΔSFT to maintain spiral pattern formation to be 1.9 mN/m.

### Force provided by Marangoni convection

Based on the motion of the red PS particle (diameter 22 µm, density 1.050 g/cm^3^, volume 5.6 × 10^−15^ m^3^, weight 5.88 × 10^−9^ g) in Supplementary Movie 5, we estimated the force provided by Marangoni flow (Supplementary Calculations). The calculation results show that, for a 22-µm PS particle in the flow field at *t* = 107.8 s, the maximum Marangoni driven force (*F*_*Mmax*_) roughly equals to the maximum viscous force (0.7 nN), which is more than two orders of magnitude larger than the maximum *ma* (1.05 × 10^−6^ nN), and the difference between gravity (5.74 × 10^−2^ nN) and buoyancy (5.52 × 10^−2^ nN). The result is similar to that of a droplet with a similar size moving in a thermal gradient-induced Marangoni convection^31^. Similarly, for the particles/droplets (diameter ~ 1 µm) in the filament, the *F*_*Mmax*_ is estimated to be 0.03 nN. Then we obtained a series of *F*_*Mmax*_ by analyzing the upward motion of particles at different times. We found that *F*_*Mmax*_ decreases linearly with time, consistent with the linear decrease of angular velocity of spiral patterns with time on the surface of the PEG-SDBS solution.

Since the maximum ΔSFT (Δγ) that can be achieved in our system is 8.9 mN/m, the theoretical characteristic velocity scale Δγ/*η* is about 1 m/s, which is around three orders of magnitude larger than the maximum velocity in our system (~ 0.5 mm/s). The result indicates that the flow is driven not by the full SFT difference of 8.9 mN/m, but by a very much smaller difference (~0.05% Δγ). This is probably because the dense filament tends to sink after it reaches the surface, leading to only a very outermost portion of the filament (with a very low concentration of CaCl_2_) to touch the surface. Consequently, the surface concentration of CaCl_2_ and the associated Marangoni force are reduced, which in turn slows down the flow and causes the filament to sink even more. A quantitative boundary-layer analysis of this mechanism is given in the Supplementary Information (Calculations section).

### Liquid rope coiling

In our experiments, the motion of the rising filament beneath the upper surface often takes the form of “liquid rope coiling” (LRC). The driving force for LRC in this case is not gravity, but rather upward advection of the filament by Marangoni convection. LRC is a buckling instability that occurs because an axial compressive stress is set up in the filament by its forced motion towards the effectively impermeable upper surface.

**Fig. 8:**
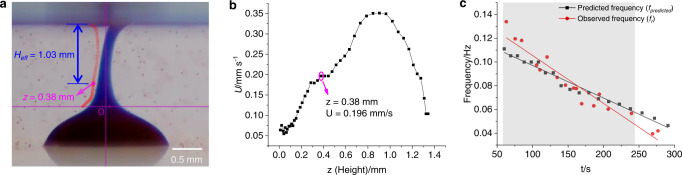
Calculation of the coiling frequency. **a **The trajectory of a PS particle near the filament moving up from the droplet to the surface of the bulk solution (102.8 to 109.8 s in Supplementary Movie 5). **b** Depth-dependent vertical velocity *U*(*z*) of a PS particle near the filament moving up from the droplet to the surface of the bulk solution (102.8 to 109.8 s in Supplementary Movie 5). **c** Coiling frequency *f*_*predicted*_(*t*) predicted from Eq. (1), where the effective velocity *U*_*0*_ and fall height *H*_*eff*_ are determined as explained in the text. For comparison, the *f*_*r*_(*t*) curve (red) obtained from the rotation of the spiral patterns in the same system is also shown.

To understand the coiling mechanism, we compared the observed coiling frequency with a theoretical prediction. Since the density of the droplet is only slightly greater than that of the bulk solution, inertia and effective gravity are both very small. The coiling is, therefore, a purely kinematical phenomenon in which the filament is forced upward against gravity by the Marangoni convection. This behavior corresponds to the viscous coiling regime, in which only viscous forces act on each material element within the filament. The expression for the coiling frequency in this regime is^28^

1 $$f=0.512\,{U}_{0}/{H}_{eff}$$

where *H*_*eff*_ is the effective height of the filament from the ejection point to the surface and *U*_*0*_ is the velocity of ejection. Expression (1) is valid for a filament ejected from a nozzle at a constant speed *U*_*0*_. To estimate this speed for our system, we measured the upward velocity *U*(*z*) of the filament as a function of height *z* above the DEX-rich droplet by tracing the motion of PS particles close to the filament in a 24 × 24 × 24 mm glass cuvette filled with PEG-SDBS solution (depth 2.5 mm) (Fig. 8a, b, and Supplementary Movie 5). The measurements showed that *U*(*z*) first increased and then decreased with increasing height (Fig. 8b). To estimate *U*_*0*_ and *H*_*eff*_, we noted that the *U*(*z*) curve has an inflexion point at *z* = 0.38 mm. Taking the distance between this point and the surface as *H*_*eff*_ (1.03 mm) (Fig. 8a) and the upward velocity as *U*_*0*_ (0.196 mm/s), we found that the frequency *f*_*predicted*_ predicted by Eq. (1) was 0.097 Hz. Similarly, a series of *H*_*eff*_ and *U*_*0*_ were obtained by tracking the upward motion of particles at different times. The values of *H*_*eff*_ and *U*_*0*_ were found to decrease with time (Supplementary Table 3). A curve *f*_*predicted*_(*t*) of predicted frequency vs. time was then obtained by applying Eq. (1). For 60 s ≤ *t* ≤ 244 s, the curve *f*_*predicted*_(*t*) agrees to within 20% with the frequency *f*_*r*_(*t*) estimated from the formation of spiral patterns (Fig. 8c), confirming that the coiling was kinematically driven by Marangoni convection. As a comparison, we also investigated the coiling behavior in the PEG-SDBS solution with a larger depth (3.4 mm) in the same cuvette (Supplementary Table 3, Supplementary Fig. 9a, b). The *f*_*r*_(*t*) curve obtained roughly coincided with that obtained using a depth of 2.5 mm (Supplementary Fig. 9c), further confirming that depth (between 1.5 and 4.8 mm) almost has no effect on the formation of spiral patterns on the surface. Both *H*_*eff*_ and *U*_*0*_ are around 1.4 times higher than those using a solution depth of 2.5 mm (Supplementary Fig. 9d, e). The corresponding curve *f*_*predicted*_(*t*) agrees to within 20% with the frequency *f*_*r*_(*t*) from 71 to 227 s (Supplementary Fig. 9f), further supporting our proposed coiling mechanism.

### Applications

#### Variation of the patterns

**Fig. 9:**
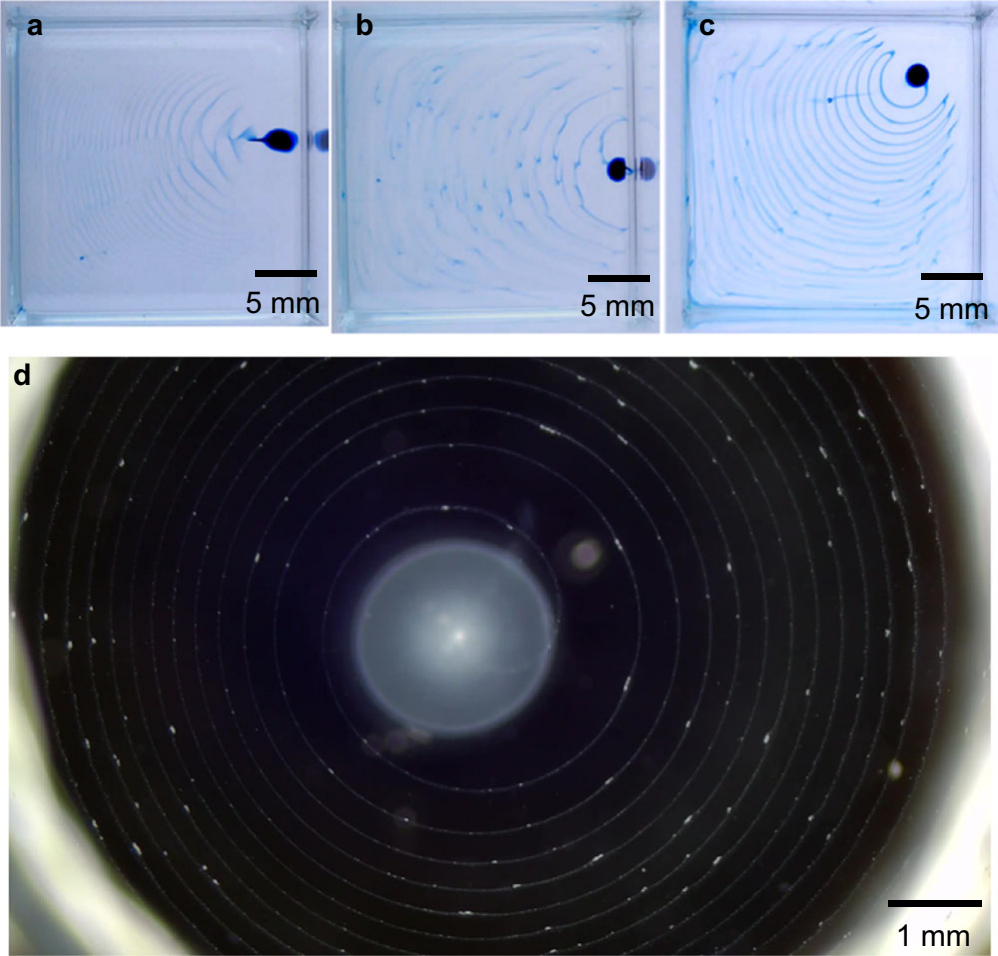
Different types of patterns. **a** mackerel-cloud, **b** half circles, and **c** crescent patterns formed by placing the DEX-MB-CaCl_2_ droplet near the wall, next to the wall, and near the corner in the cuvette, respectively. **d** Formation of PEI-PEG spiral patterns by adding PEI (0.5%)-DEX (16%)-CaCl_2_ (50 mM) droplet to the PEG-SDBS (2.5 mM) bulk solution. The photo was taken under white light illumination.

Seeing that our spiral patterns were generated by a Marangoni circulation that was approximately axisymmetric, we decided to explore how the patterns changed when the axisymmetry was broken. The simplest way to do this is to deposit the DEX-MB-CaCl_2_ droplet not at the center of the experimental tank, but instead closer to a wall or to a corner between two walls. Figure 9a–c shows the patterns thus obtained. When the droplet was near a wall, the filament remained always on the side towards the center of the tank and executed back-and-forth oscillations, producing a mackerel-cloud pattern spreading away from the wall (Fig. 9a, Supplementary Movie 11). When the droplet was placed right next to the wall, the filament went up along the wall while oscillating parallel to it, producing a pattern of spreading half-circles (Fig. 9b, Supplementary Movie 12). Finally, when the droplet was placed near a corner, the back-and-forth oscillations had a larger amplitude and produced spreading crescent patterns (Fig. 9c, Supplementary Movie 13). Our setup thus shows great potential for the fabrication of diverse patterns.

#### Fabrication of particle-based spiral patterns by chemical reactions

To vary our system, we added Poly(ethyleneimine) (PEI, 0.5% w/w) to a DEX (16% w/w)-CaCl_2_ droplet while leaving the bulk PEG-SDBS solution as before. PEI can react with PEG through hydrogen bonding. After depositing the droplet at the bottom of the bulk solution, PEI-PEG particles formed on the interface between the two phases. The particles exhibited white-blue fluorescence under white light illumination (Fig. 9d), whereas no fluorescence was observed without PEI in the droplet. The particles were quickly transferred to the surface of the bulk solution to form Fermat’s spiral patterns (Fig. 9d, Supplementary Movie 14). However, our attempt to generate fibers by increasing the concentration of PEI (e.g., 5%) did not work. This was because a PEI-PEG film was formed at the droplet surface immediately when a PEI (5%)-DEX (16%)-CaCl_2_ droplet was added to the bulk solution, preventing the further transfer of CaCl_2_ from the droplet to the bulk solution. Work on the fabrication of fibers such as gel films and nylons is ongoing.

In this work, we have reported a unique method to create solutal Marangoni convection by the interaction between the salt CaCl_2_ and the surfactant SDBS in an ATPS. A bottom-up mass transfer process accompanied by coiling behavior was realized by this Marangoni convection. The driving force of the coiling was found to be Marangoni convection rather than gravity, which was verified by theoretical calculations. The coiling resulted in the formation of Fermat’s spiral patterns on the surface of the bulk solution. The system was in a non-equilibrium state due to the transfer of CaCl_2_, but the output was highly ordered spiral patterns. The results may open up a path to the construction and control of simple non-equilibrium systems in ATPSs based on mass transfer. In our system, the automatic transport of materials from droplet in the bulk solution to the air-water surface occurs in a highly ordered way. This feature makes it possible to develop methods to fabricate fibers by utilizing non-equilibrium systems. The system has great potential in applications such as automatic fiber fabrication, cargo transport and drug delivery.

## Methods

### Materials

Polyethylene glycol 35k, sodium dodecylbenzenesulfonate, methylene blue, polyethylenimine (branched), and Rhodamine 6G were purchased from Sigma-Aldrich. Calcium chloride was purchased from Aladdin Biochemical Technology Co., Ltd. (Shanghai, China). Dextran 500k was purchased from Macklin Chemistry Co., Ltd. (Shanghai, China). Polystyrene particles (diameter 22 μm) were purchased from Huge Biotechnology Co., Ltd. (Shanghai, China). Hollow glass beads (diameter 8–12 μm) were purchased from TSI Incorporated (Minnesota, USA). Calcium colorimetric assay kit was purchased from Beyotime Biotechnology (Shanghai, China).

### Measurements and data analysis

The density of the ATPS solutions was measured by a Kruss DS 7800 density meter (accuracy of the instrument:  ± 0.001 g/mL). The viscosity of the ATPS solutions was measured by an Anton Paar MCR 302 rheometer (accuracy:  ± 0.01 mPa s). The osmolarity of the ATPS solutions was measured by an Advanced Instruments model 3320 osmometer (accuracy: ± 1 mOsm/kg). The surface tension was measured by the Wilhelmy plate method using a force tensiometer (DCAT 25, DataPhysics) (accuracy: ± 0.01 mN/m). All movies and photos were obtained with a Canon 520D camera, a Photron FASTCAM SA4 high-speed camera, a Leica DM IL LED fluorescence microscope and a Zongyuan ZY-HDMI2800 digital microscope. FT-IR spectra were recorded on a PerkinElmer Spectrum Two FT-IR spectrophotometer. NMR spectra were recorded on a Bruker Avance 600 spectrometer. All the experiments and measurements were carried out at room temperature unless specified. All the curves were fitted by using OriginPro 2018 data analysis and graphing software. The movement of the PS particles was tracked by ImageJ using the TrackMate plugin. The upward motion velocity of the PS particles was obtained by Tracker 6 (Douglas Brown, Open Source Physics).

### Preparation of ATPS

After dissolution of PEG 35k (4% w/w) and DEX 500k (8% w/w) in deionized water, centrifuging of the solution and letting it stand overnight, the solution separated into two layers with similar volume (Supplementary Fig. 1): an upper PEG-rich layer (~8% w/w, density 1.009 g/cm^3^) with a tiny amount of DEX ( < 0.005% w/w), and a lower DEX-rich layer (~16% w/w, density 1.053 g/cm^3^) with a small amount of PEG ( < 0.01% w/w)^21^. The PEG-rich layer was used as the bulk solution after the addition of SDBS (2.5 or 7 mM). The DEX-rich solution with CaCl_2_ served as the source of droplets.

### Generation of spiral patterns

Typically, 1 μL of DEX-rich solution with CaCl_2_ (50 mM) and methylene blue (1%) were deposited by an autopipette at the bottom of a cell (15.8 mm in diameter) filled with 0.5 mL of PEG-SDBS (2.5 mM) solution (depth 2.5 mm). After the pipette was withdrawn, a blue filament rose from the droplet to the surface of the bulk solution to generate spiral patterns.

### Characterization of the filament

We used proton nuclear magnetic resonance (^1^H NMR) spectroscopy (Supplementary Figs. 10–12), Fourier-transform infrared (FT-IR) spectroscopy (Supplementary Fig. 13), and calcium colorimetric assay to investigate the composition of the filament. Since the amounts of materials contained in the blue filament were relatively small, it was almost impossible to collect the particles from the filament directly. We collected the precipitates generated by adding CaCl_2_ (100 mM) to the PEG-SDBS solution (7 mM). To simplify the characterization, MB was not used in the solution. Typically, 0.8 mL of CaCl_2_ (5 M) solution was added to 38.6 mL of PEG-SDBS solution (7 mM). The mixture was shaken for 15 min, followed by a centrifuge for 30 min. The white solid was obtained by washing three times with cold water and freeze-dried for 3 days (39.7 mg).

#### ^1^H NMR

Ca(DBS)_2_ (yield ~40%): ^1^H NMR (600 MHz, DMSO-*d*_6_) *δ* 7.60-7.45 (m, 2H), 7.19-7.04 (m, 2H), 1.68-1.41 (m, 4H), 1.29-0.94 (m, 16H), 0.88-0.73 (m, 5H); PEG 35k: ^1^H NMR (600 MHz, DMSO-*d*_6_) *δ* 3.50 (s, 4n H). According to the NMR data, the mole ratio and weight ratio between Ca(DBS)_2_ (mw 690) and PEG (mw 35000) in the precipitates are calculated to be around 700:1 and 14:1, respectively (Supplementary Fig. 10).

#### Calcium colorimetric assay

The calcium colorimetric assay kit solution contains *o*-cresolphthalein complexone, which can bind Ca^2+^ to form calcium-cresolphthalein complexone, with a characteristic absorbance at 575 nm. 4.2 mg of the above white solid was dissolved in 30.4 × 2 mL of H_2_O. 50 µL of the resulting solution was added to 150 µL of the calcium colorimetric assay kit solution. The UV absorbance of the resulting solution at 575 nm was measured. The whole procedure was repeated three times. The absorbance was tested to be 0.46 ± 0.02. As a control, the UV absorption of the standard CaCl_2_ solution (0.1 mM) was tested to be 0.46 ± 0.01. So, the concentration of Ca^2+^ in the solution of the white solid is also around 0.1 mM. Accordingly, the molecular weight of the white solid is estimated to be 690, the same as that of Ca(DBS)_2_. The result further indicates that the white solid is mainly Ca(DBS)_2_.

#### FT-IR

The white solid: FT-IR *ν*/cm^–1^ 2957, 2924, 2854, 1651, 1602, 1465, 1186, 1131, 1048, 1012, 830, 673, 606, and 581. The FT-IR spectrum of the white solid (Supplementary Fig. 13, black line) coalescences well with that of SDBS (Supplementary Fig. 13, red line), with almost no peaks for PEG observed (Supplementary Fig. 13, blue line), further confirming that Ca(DBS)_2_ is the main component in the precipitates.

## Supplementary information

Supplementary Information

Description of Additional Supplementary Files

Supplementary Movie 1

Supplementary Movie 2

Supplementary Movie 3

Supplementary Movie 4

Supplementary Movie 5

Supplementary Movie 6

Supplementary Movie 7

Supplementary Movie 8

Supplementary Movie 9

Supplementary Movie 10

Supplementary Movie 11

Supplementary Movie 12

Supplementary Movie 13

Supplementary Movie 14

## Data Availability

The data supporting the findings of this study are available within the article and its Supplementary Information, and also from the corresponding author upon request.
