# Supplementary material for: Generation of Fermat’s spiral patterns by solutal Marangoni-driven coiling in an aqueous two-phase system
Source: Nat Commun. 2022 Nov 23;13:7206. doi: 10.1038/s41467-022-34368-5 (PMC9684484; doi:10.1038/s41467-022-34368-5)
Supplement: Supplementary file 1 — Supplementary Information [file 41467_2022_34368_MOESM1_ESM.pdf]

Supplementary Information

Generation of Fermat's spiral patterns by solutal Marangoni-driven coiling in an aqueous two-phase system

Yang Xiao[1], Neil M. Ribe[2], Yage Zhang[1,3], Yi Pan[1], Yang Cao[1] & Ho Cheung Shum[1,3]**

[1]Department of Mechanical Engineering, The University of Hong Kong, Pokfulam Road, Hong Kong, China

[2]Lab FAST, University Paris-Saclay, CNRS, Bât. 530, Campus Univ, 91400 Orsay, France

[3]Advanced Biomedical Instrumentation Centre, Hong Kong Science Park, Shatin, New Territories, Hong Kong, China.

**Corresponding author: ribe@fast.u-psud.fr, ashum@hku.hk*

1 Supplementary Calculations S2

2 Supplementary Tables S5

3 Supplementary Figures S7

4 Supplementary References S16

1 Supplementary Calculations

Force provided by Marangoni convection.

Based on the motion of the red PS particles (diameter 22 μm, density 1.050 g cm^{-3}, volume 5.6×10^{-15} m^3, weight 5.88×10^{-9} g) in Supplementary Movie 5, we estimated the force provided by Marangoni flow. According to Newton's second law:

$$\sum F = F_M + F_b - G - F_v = m\,a \quad (2)$$

Where F is the sum of forces applied to the particle, including the force provided by Marangoni flow (F_M), buoyancy (F_b), gravity (G) and viscous force (F_v) (Supplementary Fig. 14a). m is the mass of the particle and a is the acceleration at y direction, and the maximum value of a is 0.18 mm/s^2 at t = 107.1 s (Supplementary Fig. 14b). So, the maximum value of ma is 1.05×10^{-6} nN.

The gravity (G) can be calculated by

$$G = m\,g = \rho\, 4/3\, \pi R^3 g \quad (3)$$

Where ρ is the density of the PS particle (1.050 g cm^{-3}), R is the radius of the particle (11 μm) and g is gravitational acceleration (9.8 m s^{-2}). So, the Gravity is 5.74×10^{-2} nN.

The buoyant force can be calculated by

$$F_b = \rho_l\, V g = \rho_l\, 4/3\, \pi R^3 g \quad (4)$$

where ρ_l is the density of PEG-SDBS solution (1.010 g mL^{-1}). So, the buoyant force is 5.52×10^{-2} nN.

The viscous force can be estimated by the Stokes equation[1]

$$F_v = 6\pi R \eta U \quad (5)$$

where η is the viscosity of PEG-SDBS solution surrounding the droplet (~ 10^{-2} Pa s), U is the velocity of this particle at y direction. As is shown in Supplementary Fig. 14c, U is changing with time and its maximum value is 0.35 mm/s at t = 107.8 s. So, the maximum viscous force for this particle is estimated to be 0.7 nN. Since F_{vmax} is more than two orders of magnitude larger than ma and $G - F_b$,

$$F_{Mmax} \approx F_{vmax} \quad (6)$$

So for the 22-μm PS particles in the flow field at t = 107.8 s, the Marangoni driving force is 0.7 nN. Similarly, for the particles/droplets (diameter ~ 1 μm) in the filament, the maximum Marangoni driving force is estimated to be 0.03 nN, according to Equations (5) and (6). Then we obtain a series of U_{max} and F_{Mmax} by analyzing the upward motion of particles at different times. We find that both U_{max} and F_{Mmax} decrease linearly with time, consistent with the linear decrease of angular velocity of spiral patterns with time on the surface of the PEG-SDBS solution. We also notice that, the position of the U_{max} point is also changing with time, which is probably resulted from the oscillation of the filament.

Similarly, we also analyze the Marangoni force in the system with a bulk solution depth of 3.4 mm. We find that particles in this system have similar $U_{max}(t)$ and $F_{Mmax}(t)$ but larger values, compared to those obtained in the system with a depth of 2.5 mm (Supplementary Fig. 14d-f).

Model problem for $CaCl_2$ concentration around a moving filament.

As the filament moves horizontally, the $CaCl_2$ it contains diffuses outward into the ambient fluid and is advected by the flow in that fluid. Let the horizontal velocity of the filament be U, and let the radius of the filament be a. The diffusivity of $CaCl_2$ in water is D. At the beginning of the experiment, the Peclet number $Pe = U\,a/D \gg 1$, which means that gradients of $CaCl_2$ are confined to a thin boundary layer of thickness $\delta \ll a$ around the filament.

For simplicity, we ignore the radius of curvature of the spiral arms and consider the filament to be an infinitely long cylinder. Supplementary Fig. 15 shows a cross-section of the filament. The concentration $c(r, \theta)$ of $CaCl_2$ in the boundary layer satisfies the boundary-layer equation

$$Pe(u\partial_\theta c + v\partial_r c) = \partial_{rr}^2 c \qquad (7)$$

where u and v are the tangential and radial components of the velocity, respectively. In (7) and henceforth, velocities are measured in units of U and distances in units of a.

Eqn. (7) can be solved analytically for the cylindrical geometry at hand. We first apply the Von Mises transformation, whereby the radial coordinate is replaced by the stream function ψ. The result is

$$Pe\partial_\theta c = \partial_\psi(u\partial_\psi c). \qquad (8)$$

Now we turn to the solution for the two-dimensional flow around an infinite cylinder at low Reynolds number, which is given on pp. 244-245 of Reference 2.[2] Expanding this solution in powers of the distance $\zeta = r - 1$ away from the cylinder's surface, we find

$$u = C\zeta \sin\theta, \psi = -1/2C\zeta^2 \sin\theta, \qquad (9)$$

where

$$C = \frac{2}{\ln(3.7/Re)} \qquad (10)$$

and $Re = Ua/v$ is the Reynolds number. Eliminating ζ from equations (9), we obtain

$$u = \sqrt{-2C\psi \sin\theta}, \qquad (11)$$

Whence (8) becomes

$$Pe\partial_\theta c = \sqrt{2C \sin\theta}\, \partial_\psi(\sqrt{-\psi}\partial_\psi c). \qquad (12)$$

Now define a new timelike variable

$$t = \frac{\sqrt{2C}}{Pe}\int_0^\theta \sqrt{\sin\theta}\, \mathrm{d}\theta = \frac{\sqrt{2C}}{Pe} G(\theta). \qquad (13)$$

Equation (12) then simplifies to

$$\partial_t c = \partial_\psi(\sqrt{-\psi}\partial_\psi c). \qquad (14)$$

At this point, we assume that the concentration on the surface of the cylinder is uniform and equal to αc_0, where c_0 is the $CaCl_2$ concentration in the interior of the filament. Equation (14) then admits a similarity solution $c = c(\eta)$ in terms of the similarity variable $\eta = -\psi/t^{2/3}$. Substituting this form into (14), we obtain

$$-\frac{2}{3}\eta c' = (\sqrt{\eta} c')' \qquad (15)$$

where primes denote derivatives with respect to η. Finally, we set $z = \sqrt{\eta}$, whereupon (15) becomes

$$\frac{\mathrm{d}^2 c}{\mathrm{d}z^2} + \frac{4}{3} z^2 \frac{\mathrm{d}c}{\mathrm{d}z} = 0. \qquad (16)$$

The solution of (16) is

$$c = A\int_0^z \exp\left(-\frac{4}{9}\xi^3\right) \mathrm{d}\xi + B \qquad (17)$$

where A and B are determined by the boundary conditions $c(0) = \alpha c_0$ and $c(\infty) = 0$. We find

$$A = -\frac{3c_s}{(9/4)^{1/3}\Gamma(1/3)} \approx -0.855\alpha c_0, B = \alpha c_0. \qquad (18)$$

Finally, the variable z is related to the original variables ζ and θ by

$$z = \left(\frac{C \sin\theta}{2}\right)^{1/2} \left(\frac{Pe}{\sqrt{2}CG(\theta)}\right)^{1/3} \zeta. \qquad (19)$$

Now we are only interested in the vertical concentration profile at $\theta = \pi/2$, where $G(\pi/2) \sim 1.198$. Moreover, because z as given by (19) varies only slightly as a function of Re, we choose $C = 0.554$ for a typical experimental Reynolds number $Re = 0.1$. We then have

$z = 0.538Pe^{1/3}\zeta.$ (20)

Supplementary Fig. 16 shows vertical profiles of $CaCl_2$ concentration above the filament for two Peclet numbers Pe = 60 and 30. We chose $\alpha = 0.5$ because diffusion of $CaCl_2$ occurs also within the filament, so that the concentration on the boundary is intermediate between zero and c_0. In constructing Supplementary Fig. 16, we first fixed $c = 0.001c_0$ at the surface for the Pe = 60 case (Supplementary Fig. 16a). As discussed in the main text, $0.001c_0$ is the order of magnitude of the concentration difference that drives the very small flow velocities (≈ 0.5 mm s^{-1}) measured in the experiments. Because Pe = 60 greatly exceeds unity, concentration gradients are mostly confined to a boundary layer of thickness $\delta \ll a$. The concentration therefore decreases rapidly with height, diminishing to $0.001c_0$ on the surface at a height $0.985a$ above the filament. Next, we turned to the Pe = 30 case and sought the depth of the filament required to yield a smaller surface concentration $c = 0.0005c_0$. That depth is $1.287a$, 30% greater than for Pe = 60.

Supplementary Fig. 16 implies a positive feedback loop in which the dense filament slowly sinks, thereby reducing the surface concentration of $CaCl_2$ and the associated Marangoni force, which in turn slows down the flow (decreasing Pe) and causes the filament to sink even more. However, this positive feedback loop in principle works equally in the other direction, with a rising filament increasing the surface concentration of $CaCl_2$ and increasing the flow speed, causing the filament to rise even more. Why then is this second alternative not observed? The answer is diffusion, which tends to equalize the surface concentration of $CaCl_2$ and reduce the driving Marangoni force. Movies of the experiments show clearly that the outer arms of the spiral become progressively more diffuse with time, which corresponds to a reduction in the surface gradients of $CaCl_2$ and a progressive slowing down of the flow. Diffusion is therefore the ultimate reason why the experimental flow is observed to slow down rather than to speed up.

2 Supplementary Tables

Supplementary Table 1 Characterization data of the aqueous two-phase system. *Density, viscosity, and osmolarity of water and the aqueous two-phase system (ATPS) solutions. Each density or osmolarity value was determined by the average of 3 measurements. Each viscosity value was determined by the average of 50 dots.*

Solution / Characterization	H_2O	**PEG**-rich phase	**PEG-SDBS** (2.5 mM)	**PEG-SDBS** (7 mM)	**DEX**-rich phase	**DEX-CaCl$_2$** (50 mM)	**DEX** (16%)	**DEX (16%)-CaCl$_2$** (50 mM)
**Density** with error /g mL^{-1}	0.997 ±0.000	1.009 ±0.000	1.010 ±0.000	1.010 ±0.000	1.053 ±0.000	1.058 ±0.000	1.051 ±0.000	1.055 ±0.000
**Viscosity** with error /mPa s	0.85 ±0.03	9.27 ±0.02	9.49 ±0.03	9.81 ±0.05	130.04 ±0.18	135.96 ±1.16	119.05 ±0.21	119.54 ±0.13
**Osmolarity** with error /mOsm kg^{-1}	0 ±0	48 ±0.5	54 ±0.5	63 ±0.5	47 ±0.5	199 ±1.2	47 ±0.9	200 ±1.7

Supplementary Table 2 Surface tension data. *Surface tension (SFT) of water, PEG-rich and DEX-rich solutions with different components. Each SFT value was determined by the average of 3 measurements.*

A. Surface tension of water, water with $CaCl_2$ (50 or 100 mM), PEG-rich solution, PEG-rich solution with $CaCl_2$ (50 mM or 100 mM) or MB (1% w/w) or SDBS (2.5 mM)-MB (1% w/w), DEX-rich solution, DEX-rich solution with SDBS (2.5 mM), and Ethanol (96% v/v), respectively.

Soluiton / Characterization	H_2O	**H$_2$O-CaCl$_2$** (50 mM)	**H$_2$O-CaCl$_2$** (100 mM)	**PEG**-rich phase	**PEG-CaCl$_2$** (50 mM)	**PEG-CaCl$_2$** (100 mM)	**PEG-MB** (1%)	**PEG-SDBS** (2.5 mM)-**MB** (1%)	**DEX**-rich phase	**DEX-SDBS** (2.5 mM)	**Ethanol** (96%)
**SFT** with error /mN m^{-1}	72.8 ±0.1	72.7 ±0.1	72.8 ±0.1	60.7 ±0.1	60.8 ±0.1	60.6 ±0.1	60.5 ±0.1	37.0 ±0.1	60.6 ±0.1	36.1 ±0.1	23.6 ±0.1

B. Surface tension of PEG-SDBS (7 mM) solution with different concentrations of $CaCl_2$.

PEG-SDBS (7 mM) solution	**c**[CaCl$_2$] (**x**)/mM	**0**	0.05	0.1	0.2	0.5	1	2.5	5	7	10	20	40	50	100
	SFT (**y**) with error /mN m^{-1}	**37.9** ±0.1	37.4 ±0.1	36.9 ±0.1	36.2 ±0.1	34.9 ±0.2	33.3 ±0.1	30.8 ±0.1	29.5 ±0.1	29.0 ±0.1	28.7 ±0.1	28.3 ±0.1	28.3 ±0.1	28.3 ±0.1	28.5 ±0.0
	**y** *vs.* log**x** equation	—	$\mathbf{y} = 33.3 - 4.6 \log\mathbf{x} - 1.1 (\log\mathbf{x})^2$ ($R^2 = 0.991$)								$\mathbf{y} = 28.4 (\pm 0.1)$ [a]				

[a]This value with error was obtained by the average of SFT values of PEG-SDBS with 10, 20, 40, 50 and 100 mM of $CaCl_2$.

C. Surface tension of SDBS (7 mM) solution with different concentrations of $CaCl_2$.

SDBS (7 mM) solution without PEG	**c**[CaCl$_2$]/mM	**0**	0.05	0.1	0.2	0.5	1	2.5	5	7	10	20	40	50	100
	SFT with error /mN m^{-1}	**33.5** ±0.1	33.0 ±0.1	32.5 ±0.1	31.8 ±0.1	30.4 ±0.1	28.7 ±0.0	27.6 ±0.1	28.0 ±0.0	28.2 ±0.1	28.4 ±0.1	28.6 ±0.1	28.6 ±0.1	28.5 ±0.1	28.6 ±0.1

D. Surface tension of PEG-SDBS (2.5 mM) solution with different concentrations of $CaCl_2$.

PEG-SDBS (2.5 mM) solution	**c**[$CaCl_2$] (**x**)/mM	**0**	0.05	0.1	0.2	0.5	1	2.5	5	10	20	50	100
	SFT (**y**) with error /mN m^{-1}	**37.5** ±0.1	35.9 ±0.1	35.0 ±0.2	33.4 ±0.1	31.7 ±0.1	30.5 ±0.1	29.3 ±0.2	28.9 ±0.1	28.5 ±0.1	28.5 ±0.1	28.5 ±0.1	28.5 ±0.1
	**y** *vs.* log**x** equation	—	**y** = 30.7 - 4.1 log**x** (R^2 = 0.993)						**y** = 28.6 (±0.2) [b]				

[b]*This value with error was obtained by the average of SFT values of PEG-SDBS with 5, 10, 20, 50 and 100 mM of $CaCl_2$.*

E. Surface tension of SDBS (2.5 mM) solution with different concentrations of $CaCl_2$.

SDBS (2.5 mM) solution without PEG	**c**[$CaCl_2$]/mM	**0**	0.05	0.1	0.2	0.5	1	2.5	5	10	20	50	100
	SFT with error /mN m^{-1}	**35.2** ±0.1	32.6 ±0.1	31.1 ±0.1	29.4 ±0.1	27.5 ±0.0	27.8 ±0.1	28.0 ±0.1	28.2 ±0.1	28.3 ±0.0	28.5 ±0.1	28.5 ±0.1	28.6 ±0.1

Supplementary Table 3 Calculation data of rotation frequency of the filament. *H_{eff}, U_0 and $f_{predicted}$ obtained by analyzing the upward motion of PS particles near the filament from the DEX-MB-$CaCl_2$ (50 mM) droplet to the surface of the PEG-SDBS (2.5 mM) bulk solution in a 24 × 24 × 24 mm glass cuvette at different times. The depth of the solution is 2.5 mm and 3.4 mm, respectively.*

Depth of PEG-SDBS solution: **2.5 mm**				Depth of PEG-SDBS solution: **3.4 mm**			
**t**	$\mathbf{H_{eff}}$	$\mathbf{U_0}$	$\mathbf{f_{predicted}}$	**t**	$\mathbf{H_{eff}}$	$\mathbf{U_0}$	$\mathbf{f_{predicted}}$
63.0	1.021	0.267	0.134	74.3	1.517	0.340	0.115
75.5	1.037	0.242	0.119	86.7	1.450	0.356	0.126
84.6	1.019	0.235	0.118	85.7	1.495	0.322	0.110
105.8	1.030	0.196	0.097	95.2	1.463	0.306	0.107
110.5	1.037	0.189	0.093	106.8	1.486	0.289	0.100
120.5	1.003	0.204	0.104	116.7	1.483	0.311	0.107
134.6	1.007	0.171	0.087	116.7	1.496	0.307	0.105
146.5	0.999	0.157	0.080	126.7	1.480	0.255	0.088
158.0	1.012	0.154	0.078	146.7	1.455	0.230	0.081
161.8	0.989	0.150	0.078	165.0	1.344	0.230	0.088
169.3	0.980	0.124	0.065	178.2	1.369	0.196	0.073
186.3	0.999	0.123	0.063	180.2	1.322	0.210	0.081
194.1	0.978	0.139	0.073	202.3	1.349	0.170	0.065
206.6	0.994	0.117	0.060	216.7	1.384	0.141	0.052
239.5	0.949	0.105	0.057	246.8	1.328	0.117	0.045
269.3	0.985	0.076	0.040	261.5	1.335	0.102	0.039
276.8	0.916	0.075	0.042	288.3	1.324	0.071	0.027

3 Supplementary Figures

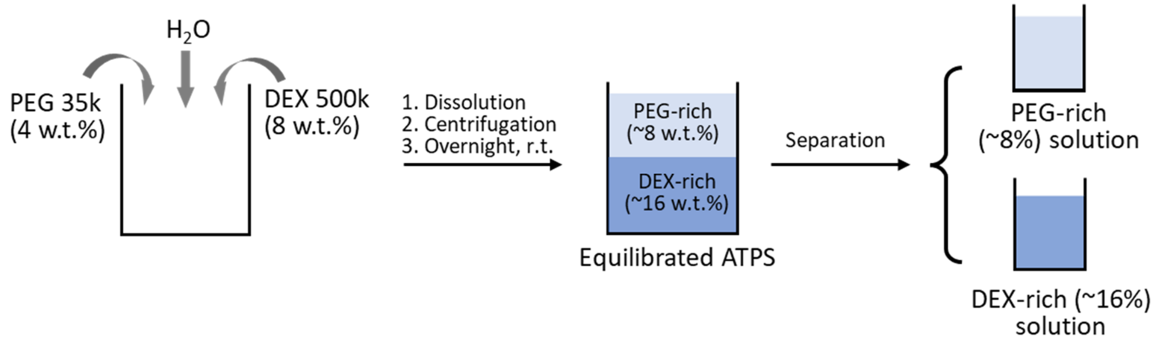

Supplementary Figure 1 Preparation of the ATPS. *Preparation of the ATPS by PEG 35k and DEX 500k.*

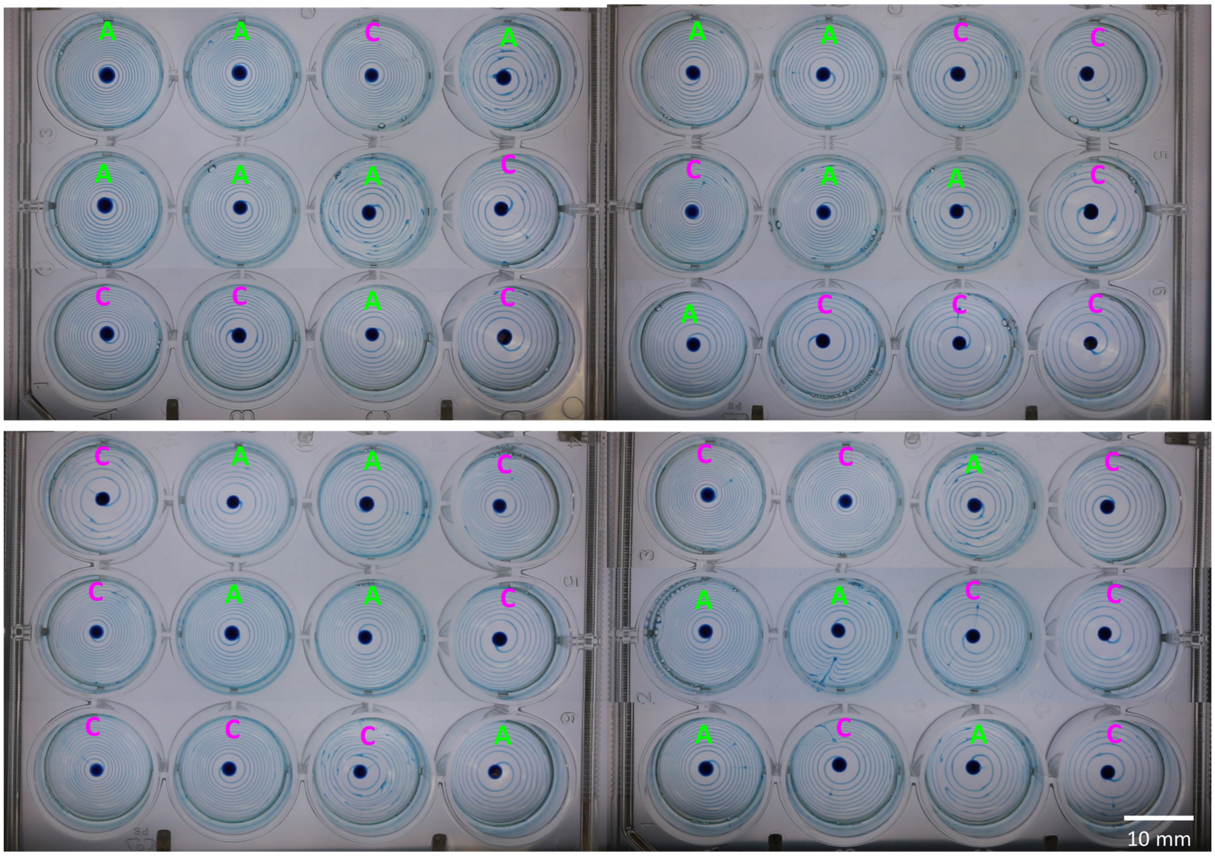

Supplementary Figure 2 Probability for the generation of clockwise and anticlockwise spiral patterns. *Probability for the formation of clockwise (C) and anticlockwise (A) spiral patterns by the addition of a 1 μL DEX-MB-$CaCl_2$ (50 mM) droplet to a cell (diameter 15.8 mm) filled with PEG-SDBS (2.5 mM) solution (depth 2.5 mm). 48 experiments were performed at the same condition, and 26 clockwise patterns (54%) and 22 anticlockwise patterns (46%) were observed, indicating the similar probability to obtain clockwise and anticlockwise patterns.*

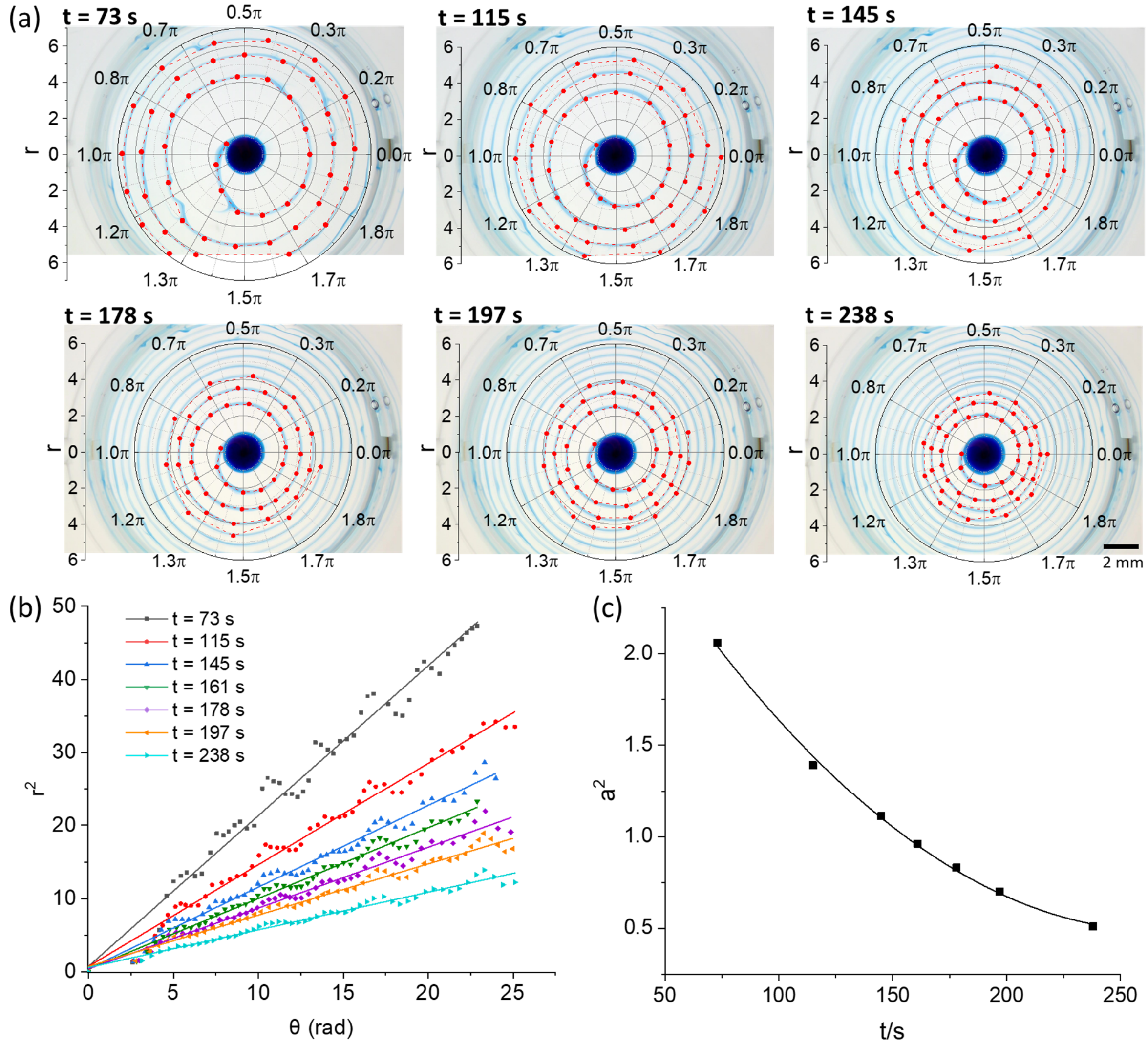

Supplementary Figure 3 Fermat's spiral patterns at different times. *(**a**) Polar coordinates (r, θ) of selected points on the spiral at t = 73, 115, 145, 178, 197, and 238 s, respectively. Images were obtained from Supplementary Movie 3. (**b**) Plot of* r^2 *vs. θ for those spirals, and (**c**)* a^2 *vs time curve.*

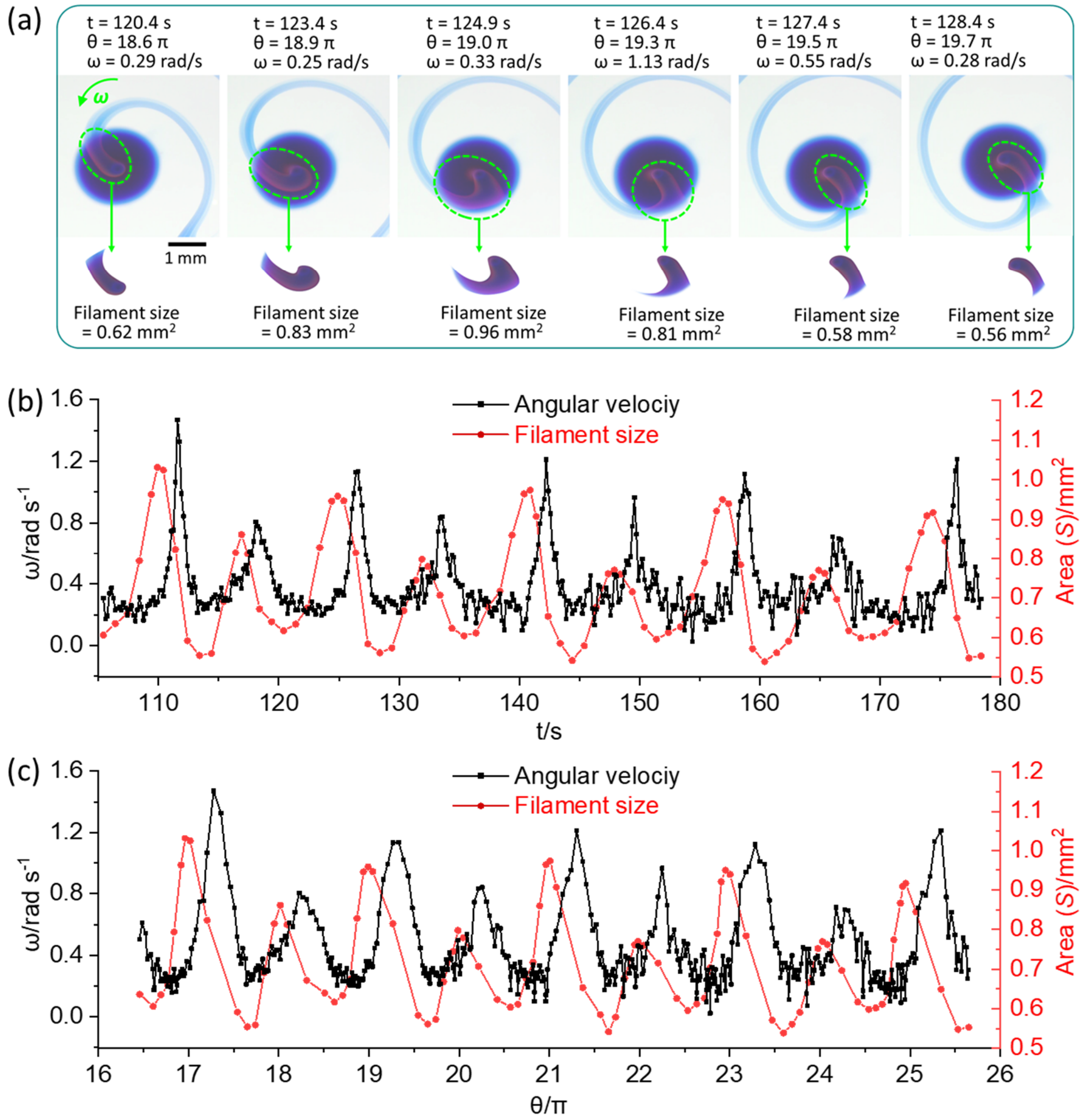

Supplementary Figure 4 Angular velocity and filament size at different times and angles. *(**a**) Angular velocity and the size of the filament which appears red near the spiral core on the surface of PEG-SDBS (2.5 mM) solution above the droplet at different angles. Images were taken from Supplementary Movie 4. (**b**) Red line and right Y-axis: filament size curve S(t) obtained by measuring the area of the filament near the spiral core on the surface of PEG-SDBS (2.5 mM) solution above the droplet at different times. Black line and left Y-axis: Angular velocity curve ω(t) obtained by tracking the rotation of the filament during spiral pattern formation by the deposition of a DEX-MB-$CaCl_2$ (50 mM) droplet to a round cell (diameter 15.8 mm) filled with PEG-SDBS (2.5 mM) solution (depth 2.5 mm) (Supplementary Movie 4). (**c**) Red line and right Y-axis: filament size curve S(θ) obtained by measuring the area of the filament near the spiral core on the surface of PEG-SDBS (2.5 mM) solution above the droplet at different angles. Black line and left Y-axis: Angular velocity curve ω(θ) obtained by tracking the rotation of the filament during spiral pattern formation by the deposition of a DEX-MB-$CaCl_2$ (50 mM) droplet to a round cell (diameter 15.8 mm) filled with PEG-SDBS (2.5 mM) solution (depth 2.5 mm) (Supplementary Movie 4).*

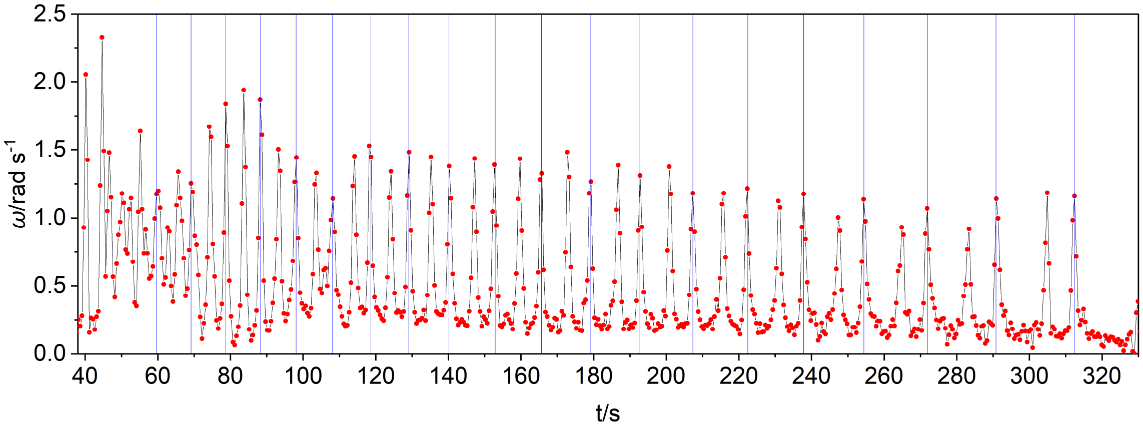

Supplementary Figure 5 The time-varying angular velocity curve obtained in a square cuvette. *The ω(t) curve obtained by tracking the rotation of the filament from top view by the deposition of a DEX-MB-$CaCl_2$ (50 mM) droplet in a glass cuvette (24 × 24 × 24 mm) filled with PEG-SDBS (2.5 mM) solution (depth 2.5 mm) (Supplementary Movie 5) using Tracker software. The ω data were recorded every 0.5 s. ω > 0 indicates counterclockwise rotation.*

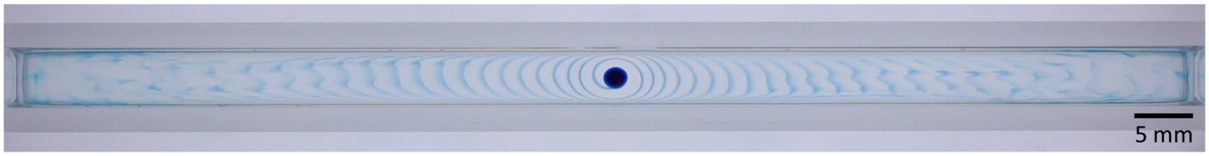

Supplementary Figure 6 Formation of spiral patterns in a narrow rectangular cuvette. *The formation of spiral patterns by adding a 1μL DEX-MB-$CaCl_2$ (50 mM) droplet to a rectangular glass cuvette (5 × 100 × 10 mm) filled with PEG-SDBS (2.5 mM) solution (depth 2.5 mm).*

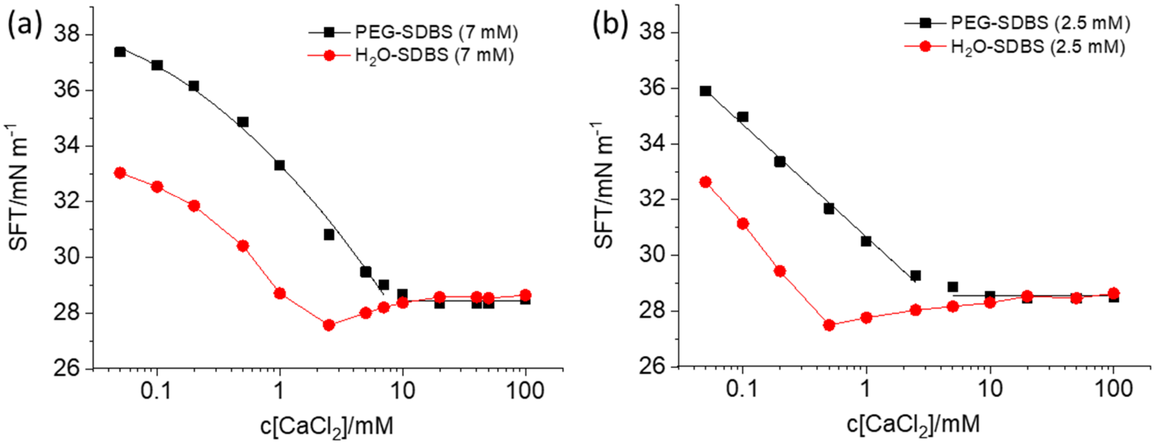

Supplementary Figure 7 SFT of PEG-SDBS and SDBS solutions with different amounts of $CaCl_2$. *(**a**) SFT vs. c[$CaCl_2$] (logarithmic scale) for PEG-SDBS (7 mM) solutions (black line) and SDBS (7 mM) solutions (red line) containing 0.05, 0.1, 0.2, 0.5, 1, 2.5, 5, 7, 10, 20, 40, 50, and 100 mM of $CaCl_2$, respectively. (**b**) SFT vs. c[$CaCl_2$] (logarithmic scale) for PEG-SDBS (2.5 mM) solutions (black line) and SDBS (2.5 mM) solutions (red line) containing 0.05, 0.1, 0.2, 0.5, 1, 2.5, 5, 10, 20, 50, and 100 mM of $CaCl_2$, respectively.*

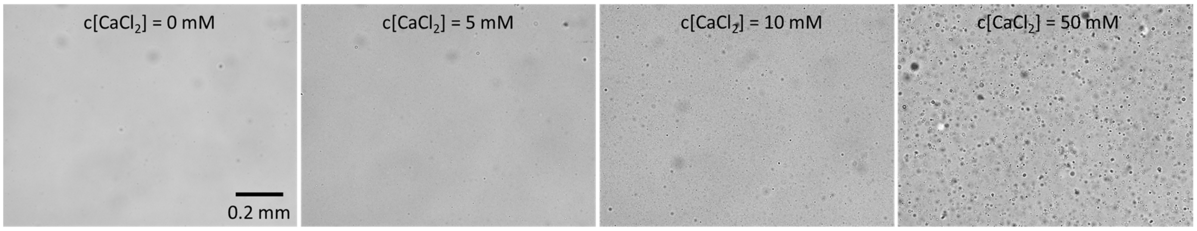

Supplementary Figure 8 Generation of precipitates by PEG-SDBS and $CaCl_2$. *Formation of precipitates in the PEG-SDBS (2.5 mM) solution with 0, 5, 10 and 50 mM of $CaCl_2$, respectively. The photos were taken using a Leica microscope.*

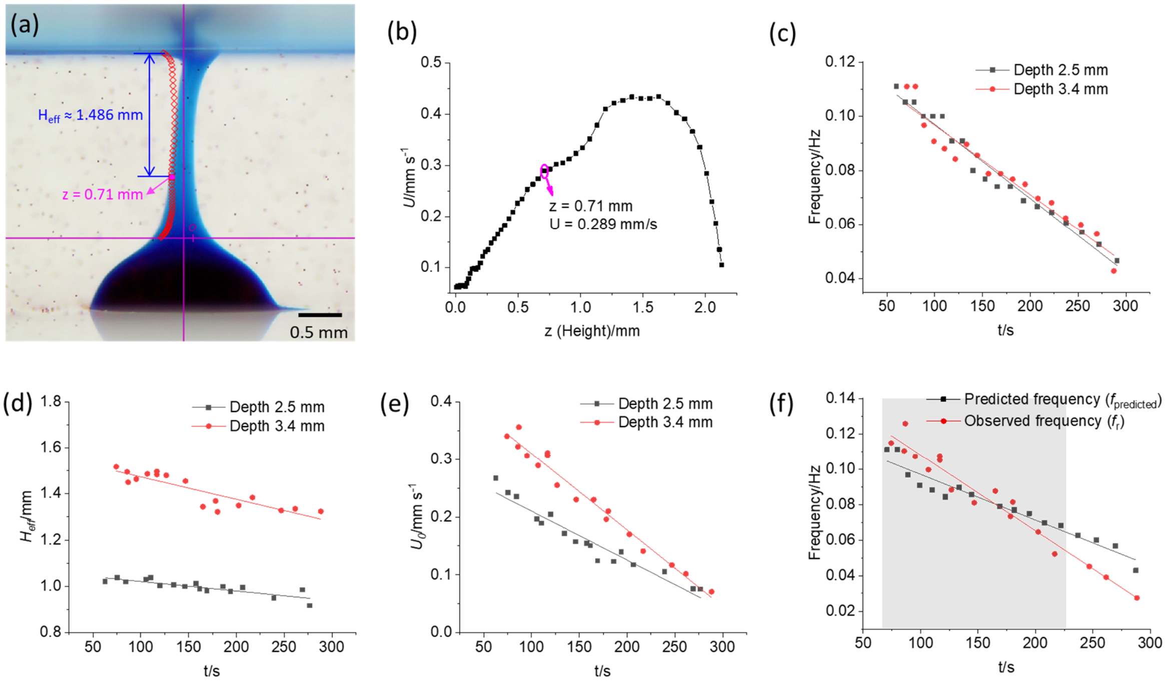

Supplementary Figure 9 Prediction of rotation frequency of the filament. ***(a)****The trajectory of a PS particle near the filament moving up from the droplet to the surface of the PEG-SDBS (2.5 mM) bulk solution (depth 3.4 mm) in a glass cuvette (24 × 24 × 24 mm) (100.3 s to 111.2 s).* ***(b)*** *Depth-dependent vertical velocity U(z) of a PS particle near the filament moving up from the droplet to the surface of the bulk solution (100.3 s to 111.2 s).* ***(c)*** *$f_r(t)$,* ***(d)****$H_{eff}(t)$, and* ***(e)*** *$U_0(t)$ curves obtained for spiral patterns in a glass cuvette (24 × 24 × 24 mm) with a solution depth of 2.5 (black line) and 3.4 mm (red line), respectively.* ***(f)*** *Coiling frequency $f_{predicted}(t)$ for the system with a depth of 3.4 mm predicted from Equation (1) in the paper, where the effective velocity U_0 and fall height H_{eff} are determined as explained in the liquid rope coiling part of the paper. For comparison, the $f_r(t)$ curve (red) obtained from the rotation of the spiral patterns in the same system is also shown. All the curves were fitted by straight lines.*

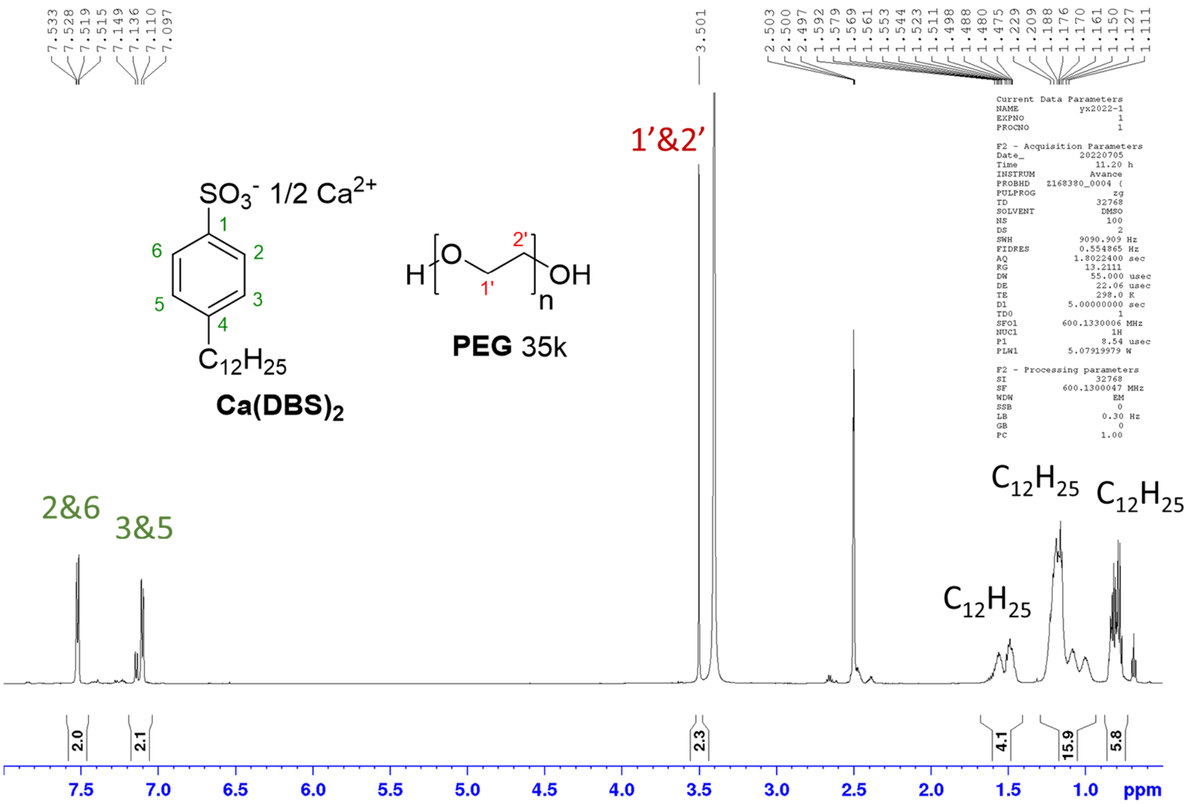

Supplementary Figure 10 ^{1}H NMR of the precipitates. *^{1}H NMR spectrum of the precipitates ($Ca(DBS)_2$ with a small amount of PEG) in DMSO-d_6.*

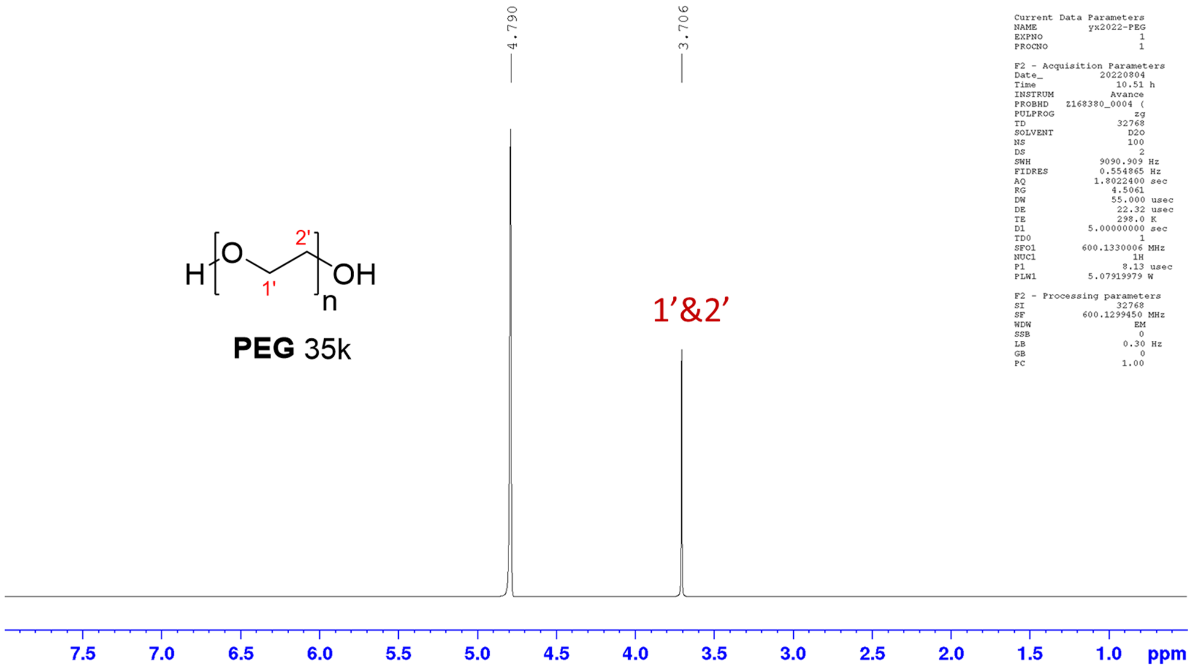

Supplementary Figure 11 ^{1}H NMR of PEG. *^{1}H NMR spectrum of PEG 35k in D_2O.*

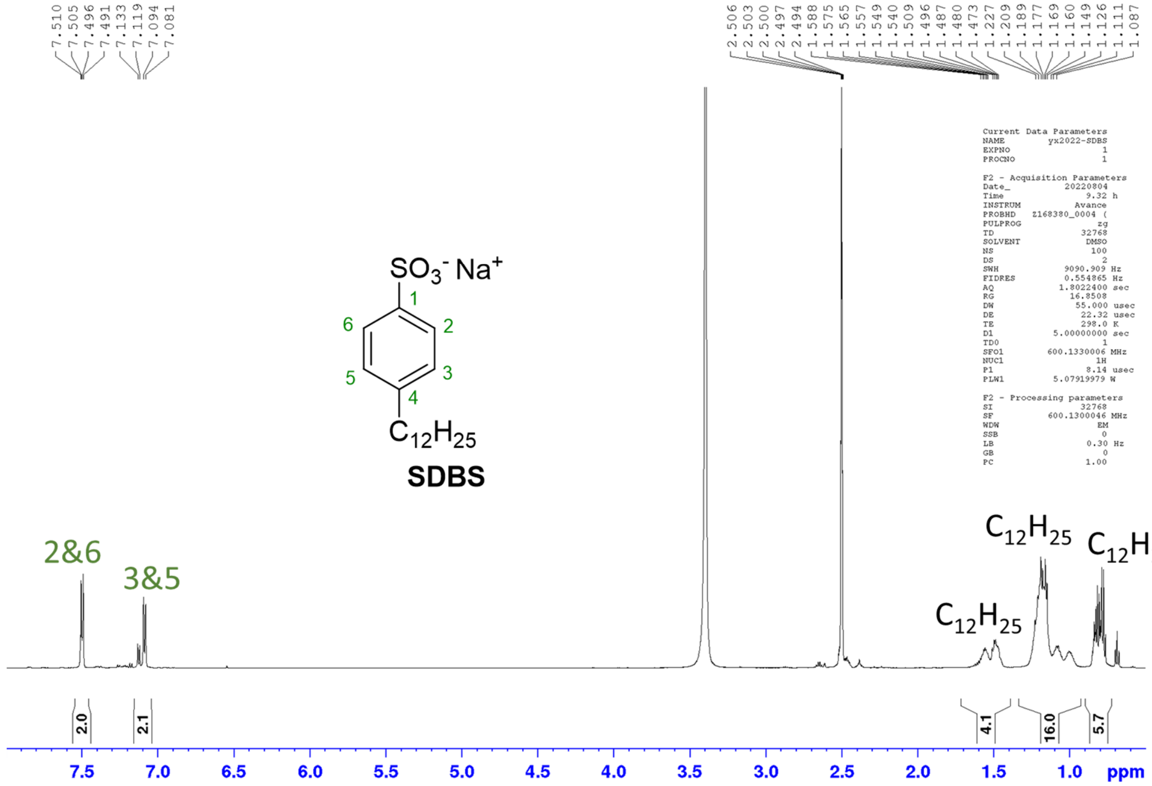

Supplementary Figure 12 ^{1}H NMR of SDBS. *^{1}H NMR spectrum of SDBS in DMSO-d_6.*

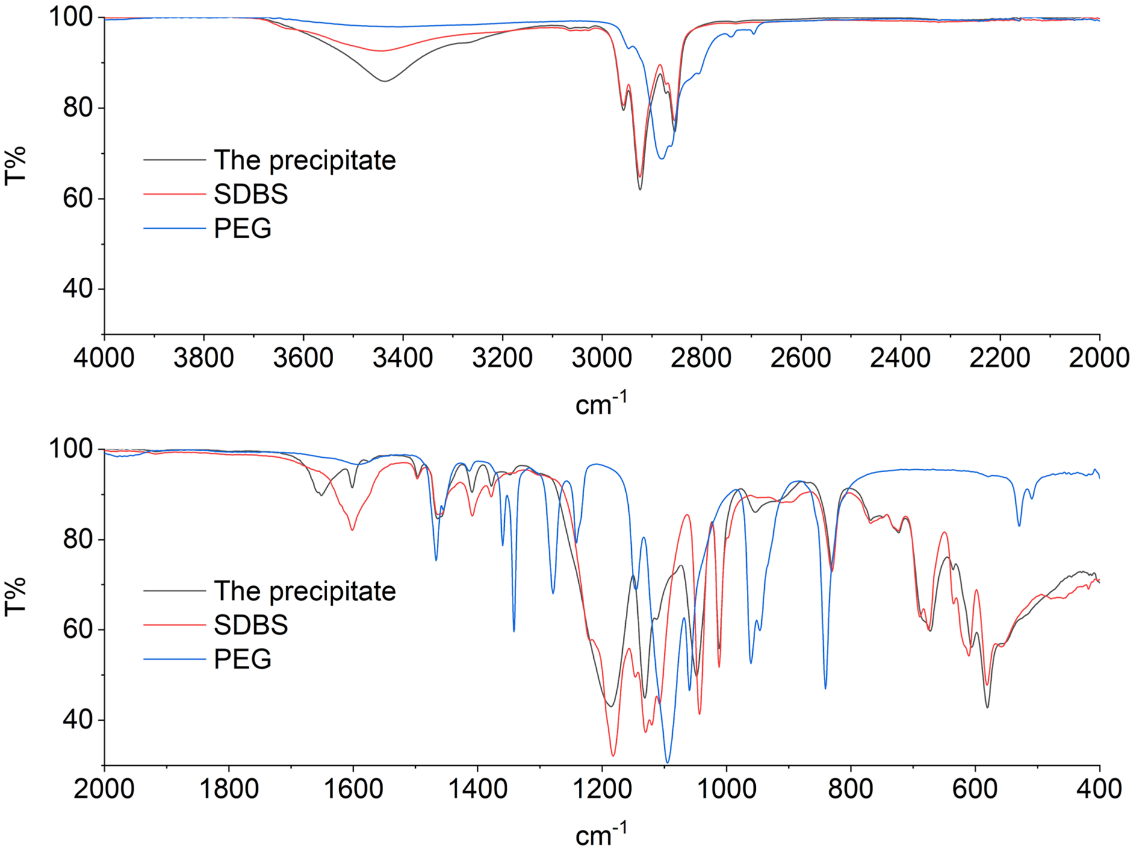

Supplementary Figure 13 FT-IR spectra. *FT-IR spectra of the precipitates ($Ca(DBS)_2$ with a small amount of PEG) (black line), SDBS (red line) and PEG (blue line).*

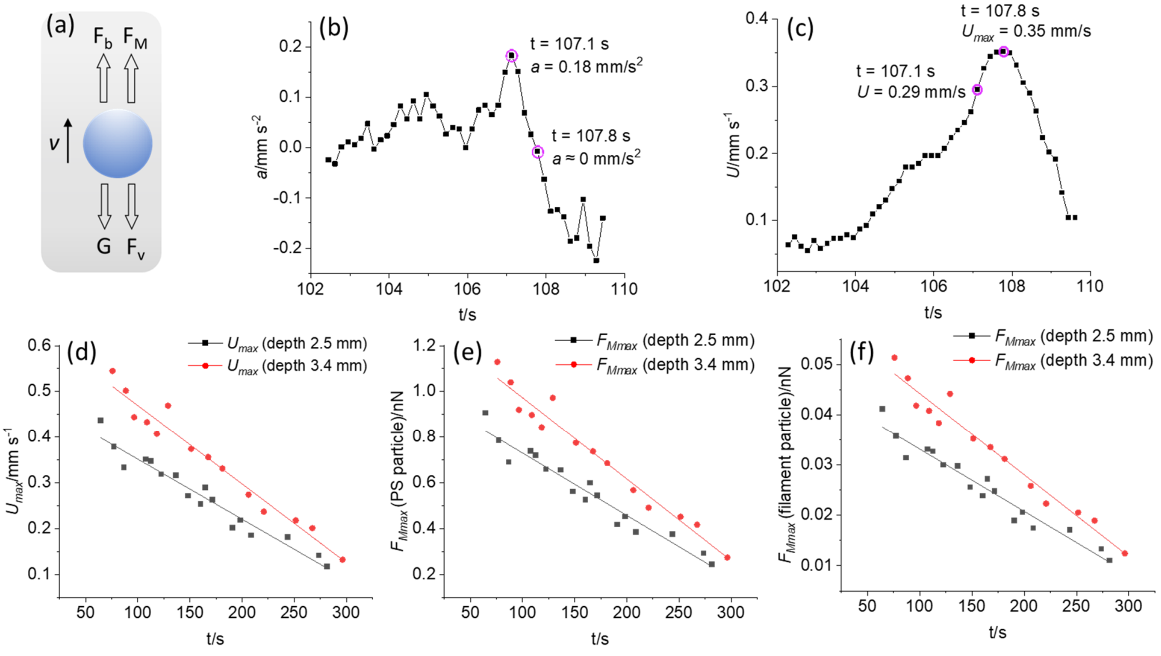

Supplementary Figure 14 Force calculation. *(**a**) Force analysis for a particle near or in the filament moving up by the Marangoni convection. (**b**) Time-dependent vertical acceleration a(t) and (**c**) time-dependent vertical velocity U(t) obtained by tracking a PS particle (diameter 22 μm) near the filament moving up from the droplet to the surface of the bulk solution by the deposition of a DEX-MB-$CaCl_2$ (50 mM) droplet in a glass cuvette (24 × 24 × 24 mm) filled with PEG-SDBS solution (depth 2.5 mm) (Supplementary Movie 5) using Tracker software. (102.8 to 109.8 s in Supplementary Movie 5). (**d**) $U_{max}(t)$, (**e**) $F_{Mmax}(t)$ (PS particles) and (**f**) $F_{Mmax}(t)$ (colloidal particles in the filament) curves obtained for Marangoni convection in a glass cuvette (24 × 24 × 24 mm) with a solution depth of 2.5 (black line) and 3.4 mm (red line), respectively.*

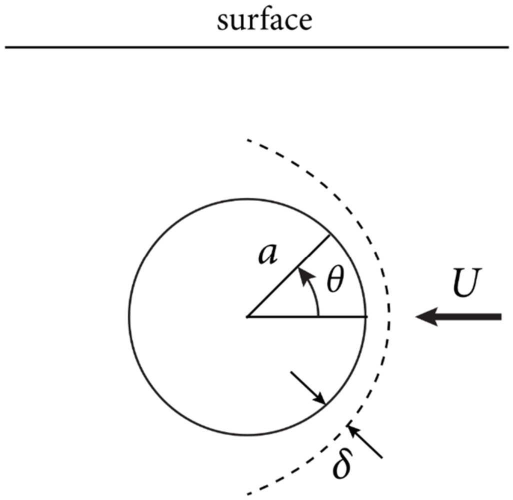

Supplementary Figure 15 Definition sketch of the model problem. *Definition sketch of the model problem for $CaCl_2$ concentration around a moving filament.*

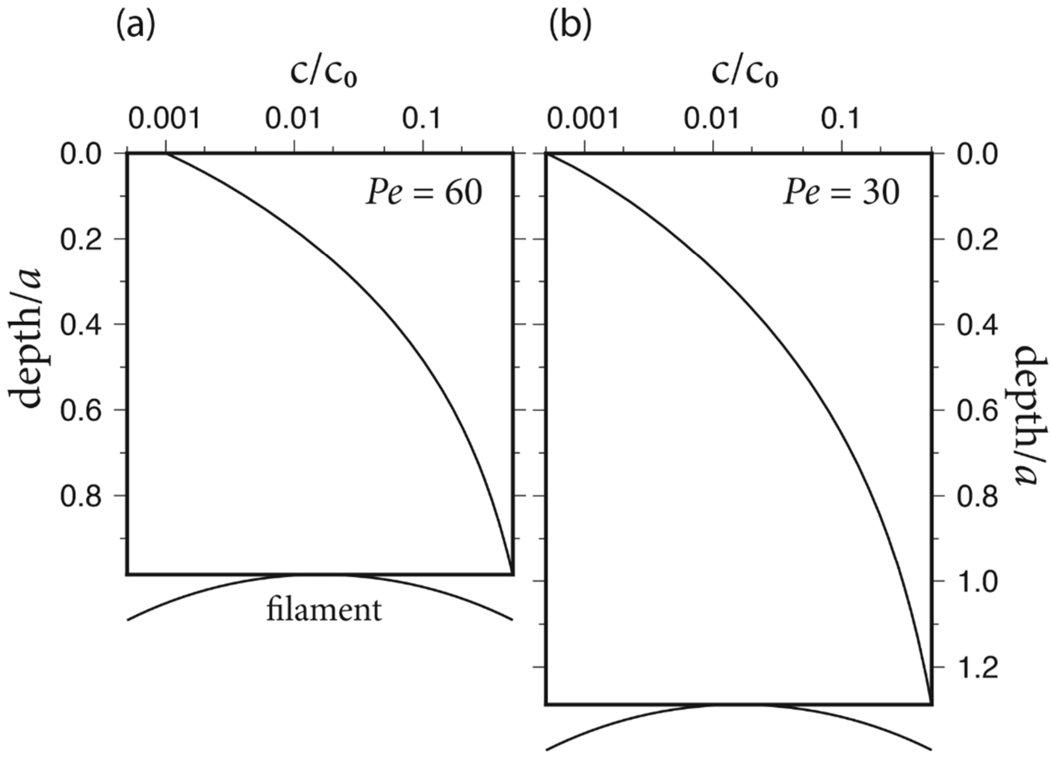

Supplementary Figure 16 $CaCl_2$ concentration above the filament for different Peclet numbers. *Vertical profiles of $CaCl_2$ concentration above the filament for Peclet number* ***(a)*** *Pe = 60 and* ***(b)*** *Pe = 30. The profiles shown are for c/c_0 = 0.001 (Pe = 60) and c/c_0 = 0.0005 (Pe = 30). The upper boundary of the filament is shown by the convex upward arcs below each panel.*

4 Supplementary References

1. Seyforth, H., Gomez, M., Rogers, W. B., Ross, J. L., Ahmed, W. W. Nonequilibrium fluctuations and nonlinear response of an active bath. *Physical Review Research* **4**, 023043 (2022).
2. Batchelor, G. K. *An introduction to fluid dynamics*. Cambridge University Press (1967).
