# Supplementary material for: Generation of Fermat’s spiral patterns by solutal Marangoni-driven coiling in an aqueous two-phase system
Source: Nat Commun. 2022 Nov 23;13:7206. doi: 10.1038/s41467-022-34368-5 (PMC9684484; doi:10.1038/s41467-022-34368-5)
Supplement: Supplementary file 2 — Description of Additional Supplementary Files [file 41467_2022_34368_MOESM2_ESM.pdf]

Descriptions of Additional Supplementary Files

Supplementary Movie 1 Movie of Marangoni convection generated by the addition of a droplet of DEX-$CaCl_2$ (0.1 M) to a solution of PEG-SDBS (7 mM) containing hollow glass beads (diameter 8-12 μm).

Supplementary Movie 2 Movie of the formation of maze-like patterns after the addition of a DEX-MB-$CaCl_2$ (50 mM, 1 μL) droplet to a round cell (diameter 15.8 mm) filled with PEG-SDBS (7 mM) solution (depth 2.5 mm, volume 0.5 mL).

Supplementary Movie 3 Movie of the formation of spiral patterns after the addition of a DEX-MB-$CaCl_2$ (50 mM, 1 μL) droplet to a round cell (diameter 15.8 mm) filled with PEG-SDBS (2.5 mM) solution (depth 2.5 mm, volume 0.5 mL).

Supplementary Movie 4 Movie of the formation of spiral patterns under white light illumination after the addition of a DEX-MB-$CaCl_2$ (50 mM, 1 μL) droplet to a round cell (diameter 15.8 mm) filled with PEG-SDBS (2.5 mM) solution (depth 2.5 mm, volume 0.5 mL). The movie was taken under white light illumination from top left.

Supplementary Movie 5 Movie of the formation of spiral patterns after the addition of a DEX-MB-$CaCl_2$ (50 mM, 1 μL) droplet to a square glass cuvette (24 × 24 × 24 mm) filled with PEG-SDBS (2.5 mM) solution (depth 2.5 mm, volume 1.46 mL). Top half of the movie: top view; bottom half: side view.

Supplementary Movie 6 Movie of Marangoni convection in the bulk solution visualized by tracking tracer particles (22 μm) in Supplementary Movie 5 (135.1-172.6 s). The green lines are the trajectories of the PS particles during 5 s. The pink dots indicate the current positions of the PS particles. The top half of the movie is the processed movie with trajectories, and the bottom half is the original movie (bottom left: side view; bottom right: top view).

Supplementary Movie 7 Movie of Marangoni convection induced by the deposition of a tiny amount of DEX-MB-$CaCl_2$ (< 0.05 μL) on the surface of a PEG-SDBS solution (2.5 mM) containing tracer particles (22 μm). The green lines are the trajectories of the PS particles during 5 s. The pink dots indicate the current positions of the PS particles. The top half of the movie is the processed movie with trajectories, and the bottom half is the original movie.

Supplementary Movie 8 Movie of the transfer of the blue filament from a DEX-MB-$CaCl_2$ droplet (1 μL) to the bulk solution surface induced by a tiny amount of DEX-MB-$CaCl_2$ (< 0.05 μL) on the surface above the droplet. The droplet was initially deposited carefully at the bottom of the PEG-SDBS (2.5 mM) bulk solution. Left half of the movie: side view; right half: top view.

Supplementary Movie 9 Movie of spontaneous diffusion of MB and $CaCl_2$ from a DEX-MB-$CaCl_2$ droplet (1 μL) that was carefully deposited at the bottom of a PEG-SDBS

(2.5 mM) bulk solution. The movie is 8 times faster than real time. Left half of the movie: side view; right half: top view.

Supplementary Movie 10 Movie of the rotation direction change of the filament from clockwise to counterclockwise by adding a PEG-SDBS (2.5 mM, 1 μL) drop with $CaCl_2$ (0.5 mM) to the outer area while the filament is rotating clockwise. The initial clockwise rotation was generated by the addition of a DEX-MB-$CaCl_2$ (50 mM, 1 μL) droplet to a square glass cuvette (24 × 24 × 24 mm) filled with PEG-SDBS (2.5 mM) solution (depth 2.5 mm, volume 1.46 mL). PS particles (diameter 22 μm) were used in the bulk solution to visualize the flow.

Supplementary Movie 11 Movie of the formation of mackerel-cloud patterns obtained by placing a DEX-MB-$CaCl_2$ (50 mM, 1 μL) droplet near the wall of a square glass cuvette (24 × 24 × 24 mm) filled with PEG-SDBS (2.5 mM) solution (depth 2.5 mm, volume 1.46 mL).

Supplementary Movie 12 Movie of the formation of half-circle patterns obtained by placing a DEX-MB-$CaCl_2$ (50 mM, 1 μL) droplet next to the wall of a square glass cuvette (24 × 24 × 24 mm) filled with PEG-SDBS (2.5 mM) solution (depth 2.5 mm, volume 1.46 mL).

Supplementary Movie 13 Movie of the formation of crescent patterns obtained by placing a DEX-MB-$CaCl_2$ (50 mM, 1 μL) droplet near the corner of a square glass cuvette (24 × 24 × 24 mm) filled with PEG-SDBS (2.5 mM) solution (depth 2.5 mm, volume 1.46 mL).

Supplementary Movie 14 Movie of the formation of PEI-PEG spiral patterns obtained by adding a PEI (0.5%)-DEX (16%)-$CaCl_2$ (50 mM) droplet to a PEG-SDBS (2.5 mM) bulk solution. Photos were taken under white light illumination.
